# Supplementary figures and images for: Integration of in situ hybridization and scRNA-seq data provides a 2D topographical map of the developing retina across species
Source: bioRxiv. 2026 Jan 4:2026.01.04.697548. Preprint. [Version 1] doi: 10.64898/2026.01.04.697548 (PMC12776276; doi:10.64898/2026.01.04.697548)

Supplementary Figure 1. Quantification of RNA-FISH signal for *Fgf8*

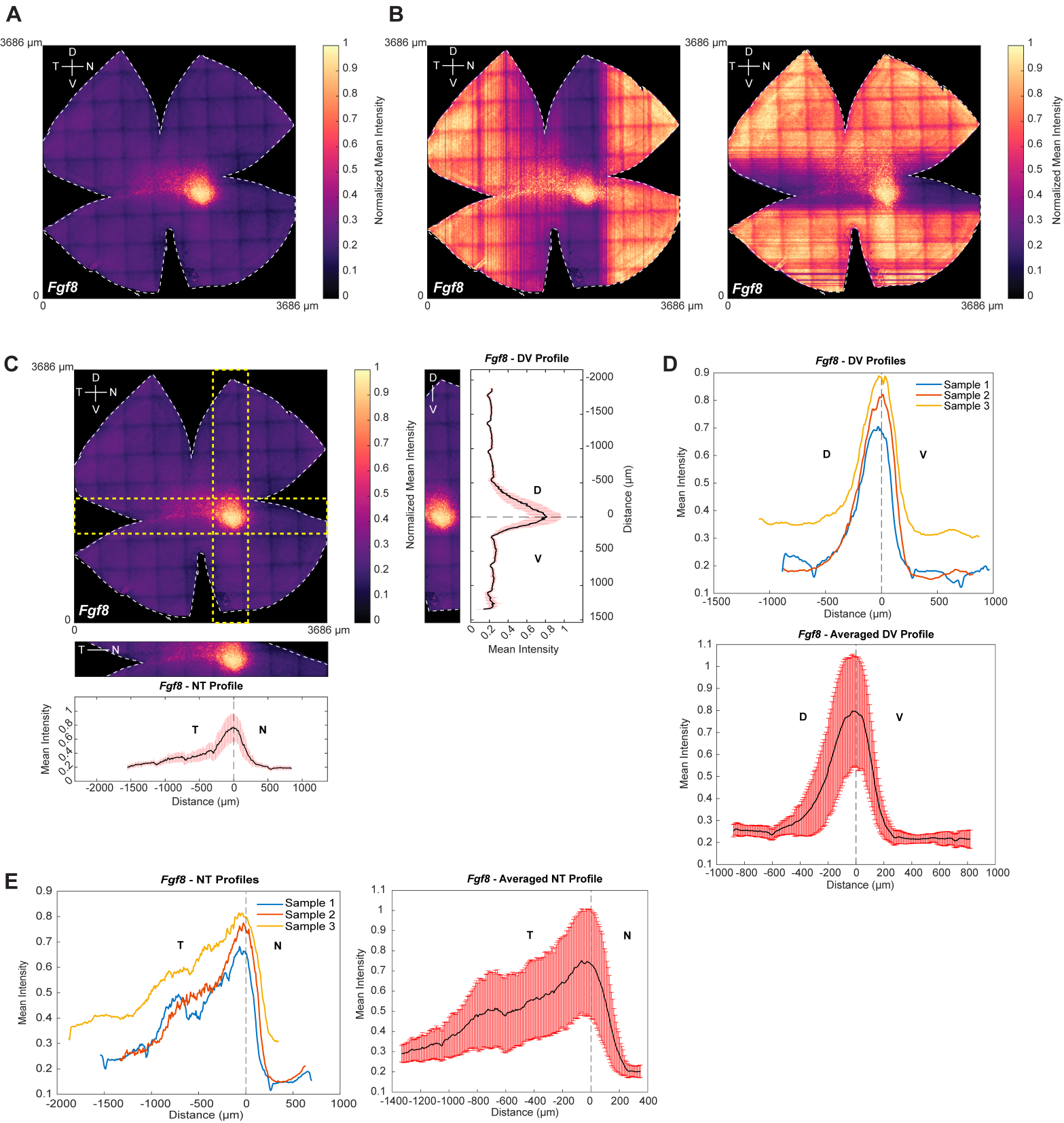

Supplement: Supplement 4 — Figure S1. Quantification of RNA-FISH signal for Fgf8 (A) Normalized mean intensity heatmap showing Fgf8 expression. (B) Column- and row-normalized intensity maps for Fgf8. (C) Spatial intensity mapping and 1D expression profiling of Fgf8. Normalized mean intensity profiles of Fgf8 along DV and NT axes are shown with the origin set at the center of the Fgf8 spot. The x-axis shows distance (μm) from the center of the Fgf8 expression spot. The y-axis shows normalized mean fluorescence intensity, calculated from RNA-FISH signal. To generate the DV and NT profiles (1D intensity profiles), a 500-μm-wide strip was extracted along the DV or NT axis, and fluorescence values were averaged across the width of the strip at each position along the axis. Black lines denote the mean normalized intensity; red lines represent the standard deviation (SD). (D, E) Spatial expression profiles of Fgf8 across three HH25/26 retinas. DV and NT profiles for mean intensity profiles were generated for each sample (N = 3) after alignment to their respective origins. The averaged DV and NT profiles are shown across the three replicates. Averaged DV and NT profiles were plotted from these 3 samples. Black lines denote the mean normalized fluorescence intensity; red lines represent the standard error of the mean (SEM). Profiles were calculated from N = 3 biological replicates. HH, Hamburger and Hamilton; D, Dorsal; V, Ventral; N, Nasal; T, Temporal. [file media-4.pdf]

Supplementary Figure 2. Quantification of RNA-FISH signal for RA pathway genes

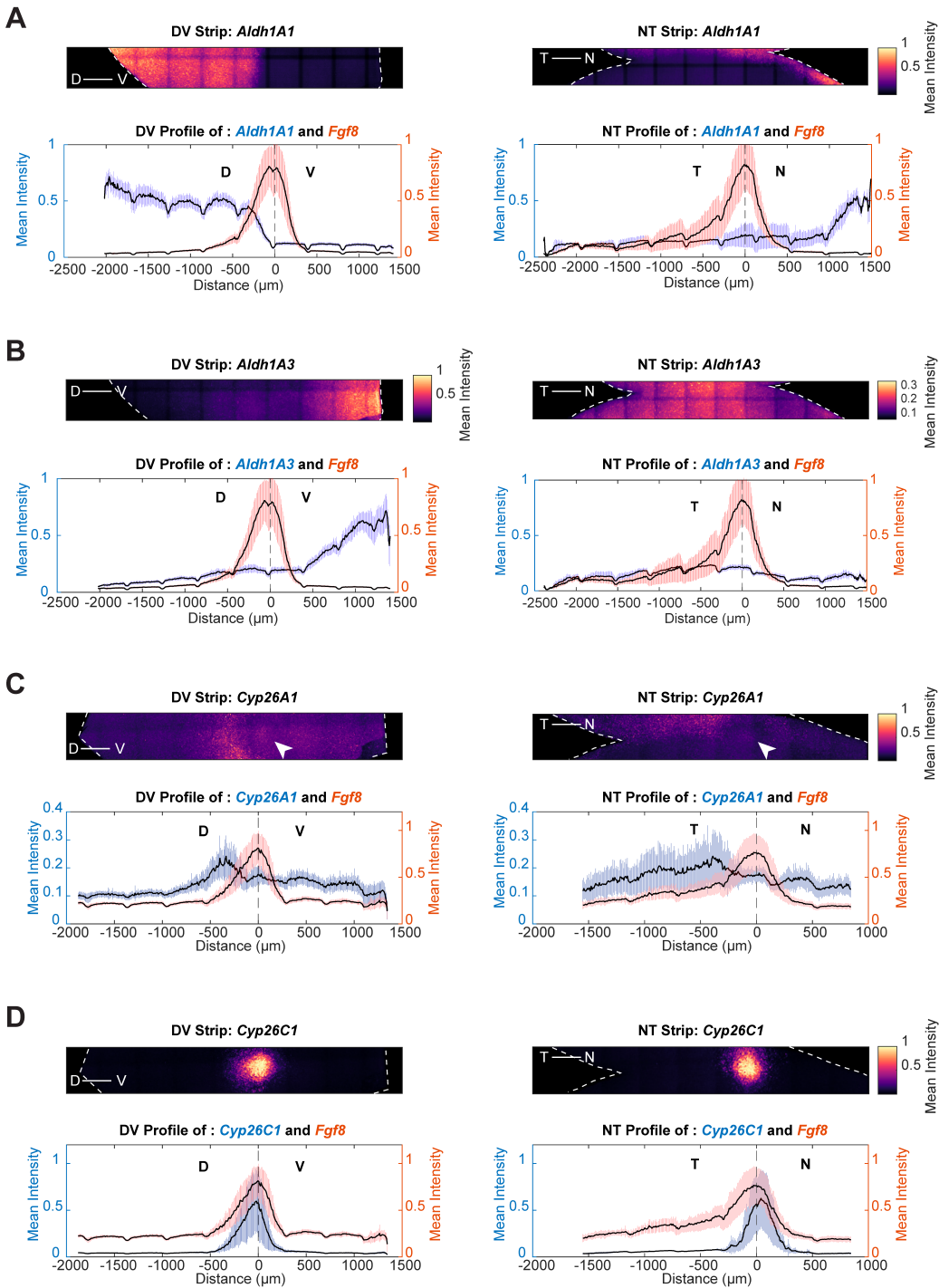

Supplement: Supplement 5 — Figure S2. Quantification of RNA-FISH signal for RA pathway genes relative to Fgf8 Normalized mean intensity profiles of RA pathway genes along DV and NT axes with the origin set at the center of the Fgf8 spot. From each origin, 500 μm wide strips were extracted along the x- and y-axes, and mean intensity values were computed across 1D cross-sections to create graphs of relative gene expression. Black lines denote the mean normalized fluorescence intensity; red/blue lines represent the standard deviation (SD). DV and NT mean intensity profiles for (A) Aldh1A1 and Fgf8 (B) Aldh1A3 and Fgf8 (C) Cyp26a1 and Fgf8 (D) Cyp26c1 and Fgf8. White arrow highlights the transient “bull’s-eye” pattern of Cyp26a1, which aligns with the Fgf8 peak along the DV and NT axis. HH, Hamburger and Hamilton; D, Dorsal; V, Ventral; N, Nasal; T, Temporal. [file media-5.pdf]

Supplementary Figure 4. Quantification of RNA-FISH signal for early DV patterning genes

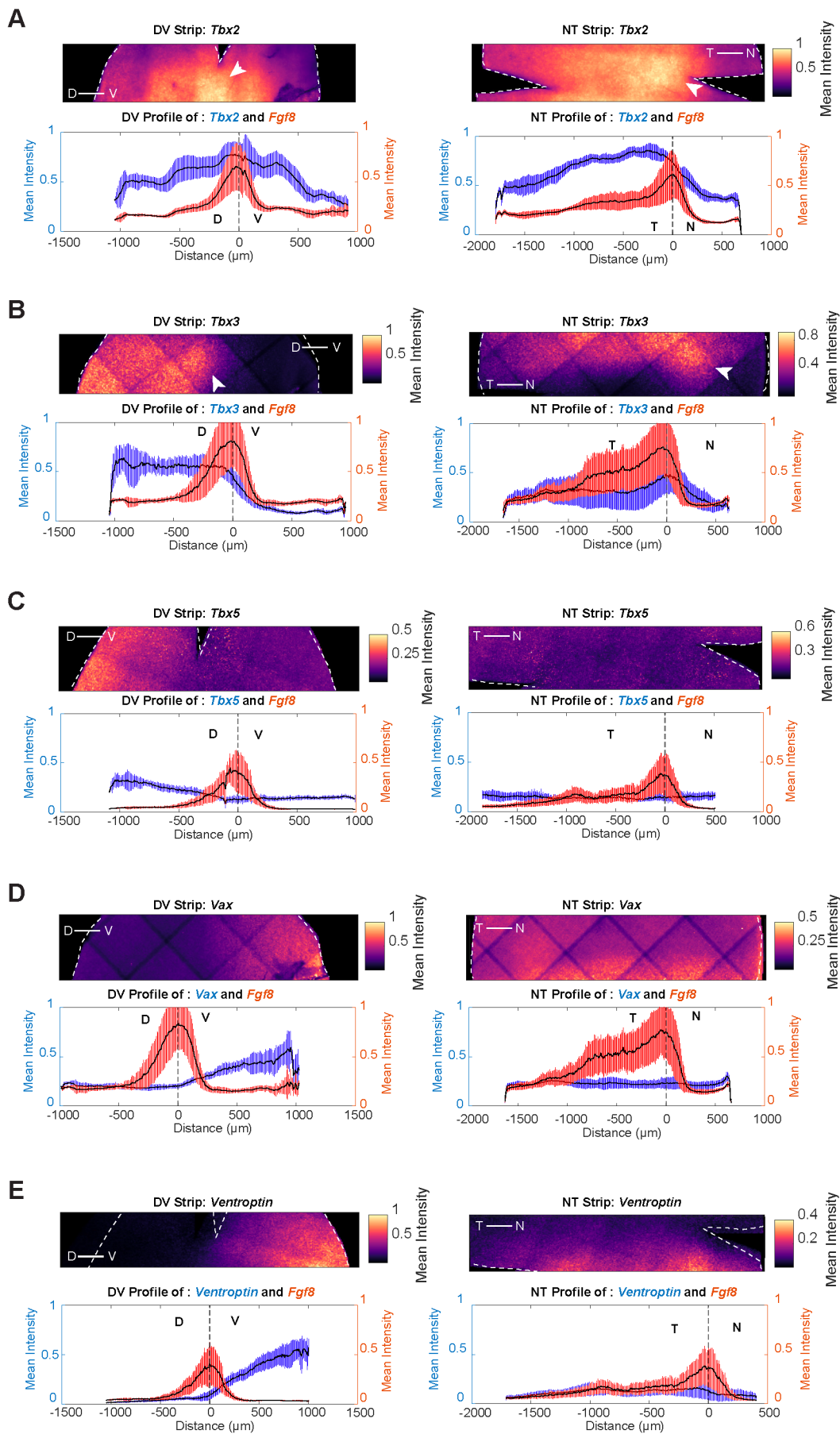

Supplement: Supplement 7 — Figure S4. Quantification of RNA-FISH signal for DV genes relative to Fgf8 Normalized mean intensity profiles of DV genes along DV and NT axes with the origin set at the center of the HAA (Fgf8 spot). From each origin, 500 μm wide strips were extracted along the x- and y-axes, and mean intensity values were computed across 1D cross-sections to create graphs of relative gene expression. Black lines denote the mean normalized fluorescence intensity; red/blue lines represent the standard deviation (SD). DV and NT mean intensity profiles for (A) Tbx2 and Fgf8 (White arrow highlights region of peak Tbx2 expression, which is placed right next to the Fgf8 spot along the DV and NT axis) (B) Tbx3 and Fgf8 (White arrow highlights region of peak Tbx3 expression, which aligns with the Fgf8 peak along the DV and NT axis) (C) Tbx5 and Fgf8, (D) Vax and Fgf8, and (E) Ventroptin and Fgf8. HH, Hamburger and Hamilton; D, Dorsal; V, Ventral; N, Nasal; T, Temporal. [file media-7.pdf]

Supplementary Figure 5. Expression domains of early DV patterning genes relative to *Fgf8* at HH25/26

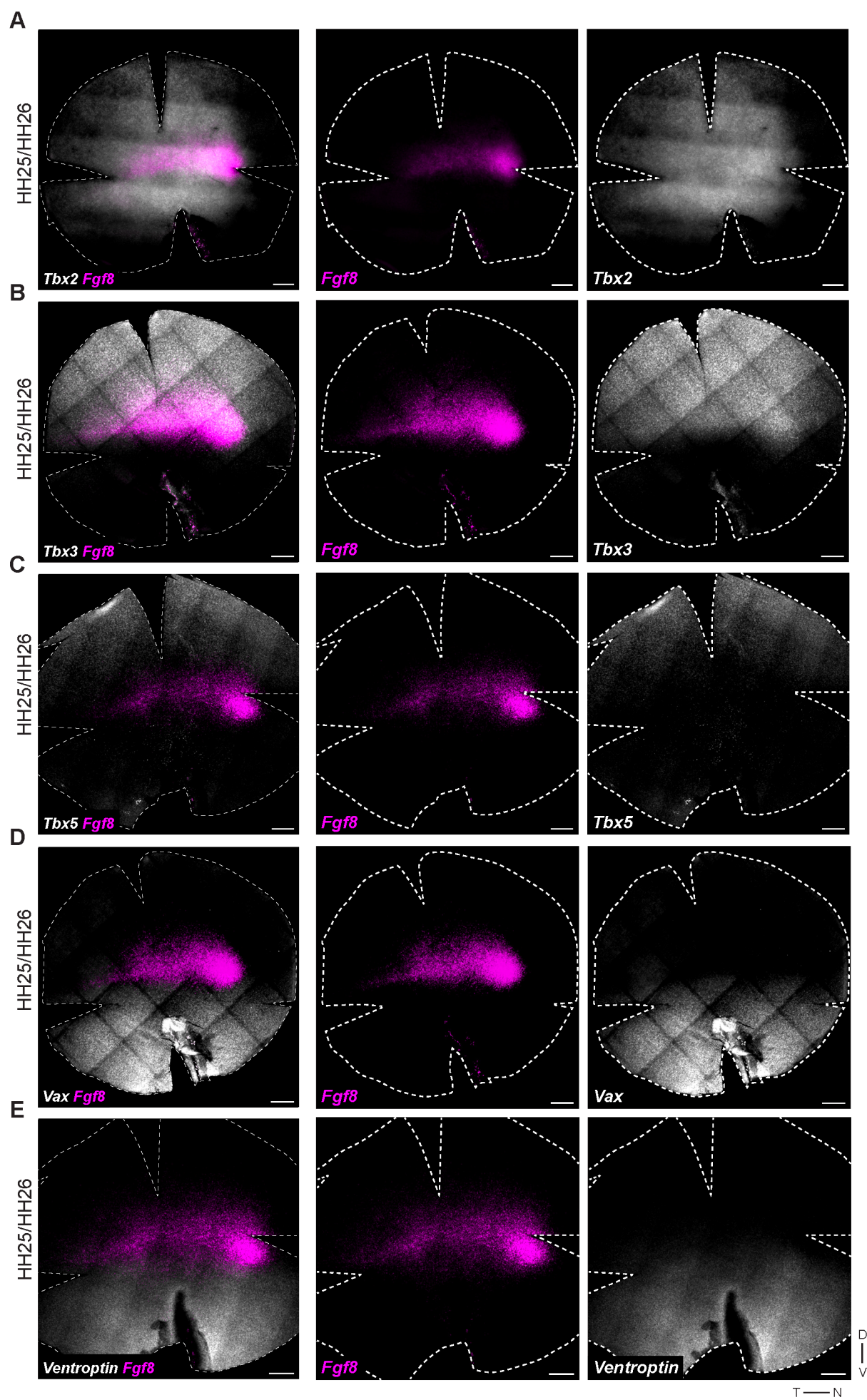

Supplement: Supplement 8 — Figure S5. Expression domains of DV genes relative to Fgf8 at HH25/26 Multiplexed RNA-FISH was performed on HH25/HH26 retinal whole mounts for (A) Fgf8 and Tbx2; (B) Fgf8 and Tbx3; (C) Fgf8 and Tbx5; (D) Fgf8 and Vax; (E) Fgf8 and Ventroptin. Each panel shows merged images and corresponding single-channel expression images. Dorsally patterned genes (Tbx2, Tbx3, and Tbx5); Ventrally patterned genes (Vax and Ventroptin); Scale bars, 200 μm. HH, Hamburger and Hamilton; D, Dorsal; V, Ventral; N, Nasal; T, Temporal. All patterns were observed in N ≥ 3 retinas. The Tbx2 image shown here (A) is a widefield image, whereas all other panels are maximum projections of confocal z-stacks. [file media-8.pdf]

Supplementary Figure 6. Expression domains of early DV patterning genes relative to Fgf8 at HH28/29

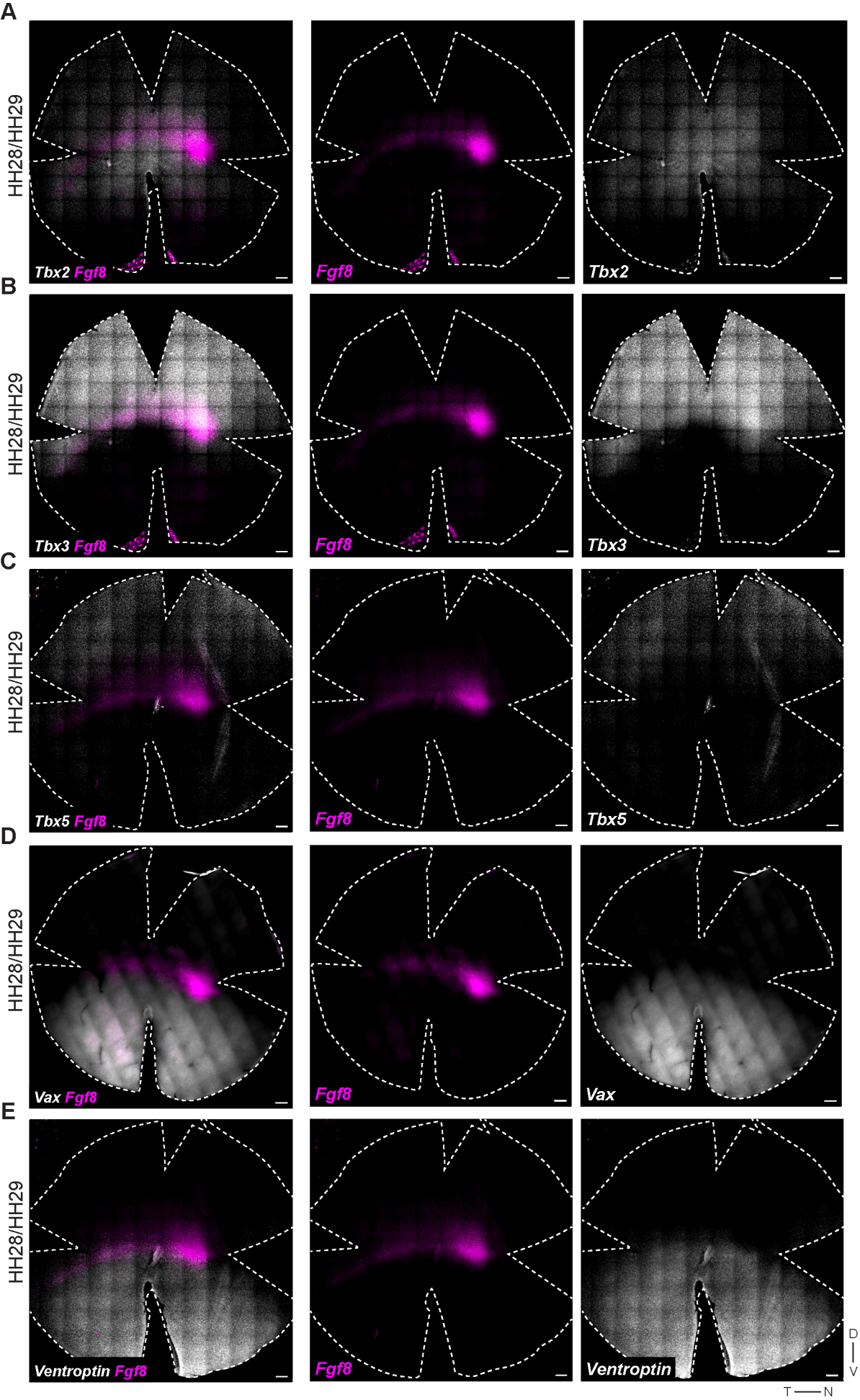

Supplement: Supplement 9 — Figure S6. Expression domains of DV genes relative to Fgf8 at HH28/29 Multiplexed RNA-FISH was performed on HH28/HH29 retinal whole mounts for (A) Fgf8 and Tbx2; (B) Fgf8 and Tbx3; (C) Fgf8 and Tbx5; (D) Fgf8 and Vax; (E) Fgf8 and Ventroptin. Each panel shows merged images, and corresponding single-channel expression images. Dorsally patterned genes (Tbx2, Tbx3, and Tbx5); Ventrally patterned genes (Vax and Ventroptin); Scale bars, 200 μm. HH, Hamburger and Hamilton; D, Dorsal; V, Ventral; N, Nasal; T, Temporal. All patterns were observed in N ≥ 3 retinas. All the images are maximum projections of confocal z-stacks. [file media-9.pdf]

Supplementary Figure 8. Quantification of RNA-FISH signal for early NT patterning genes

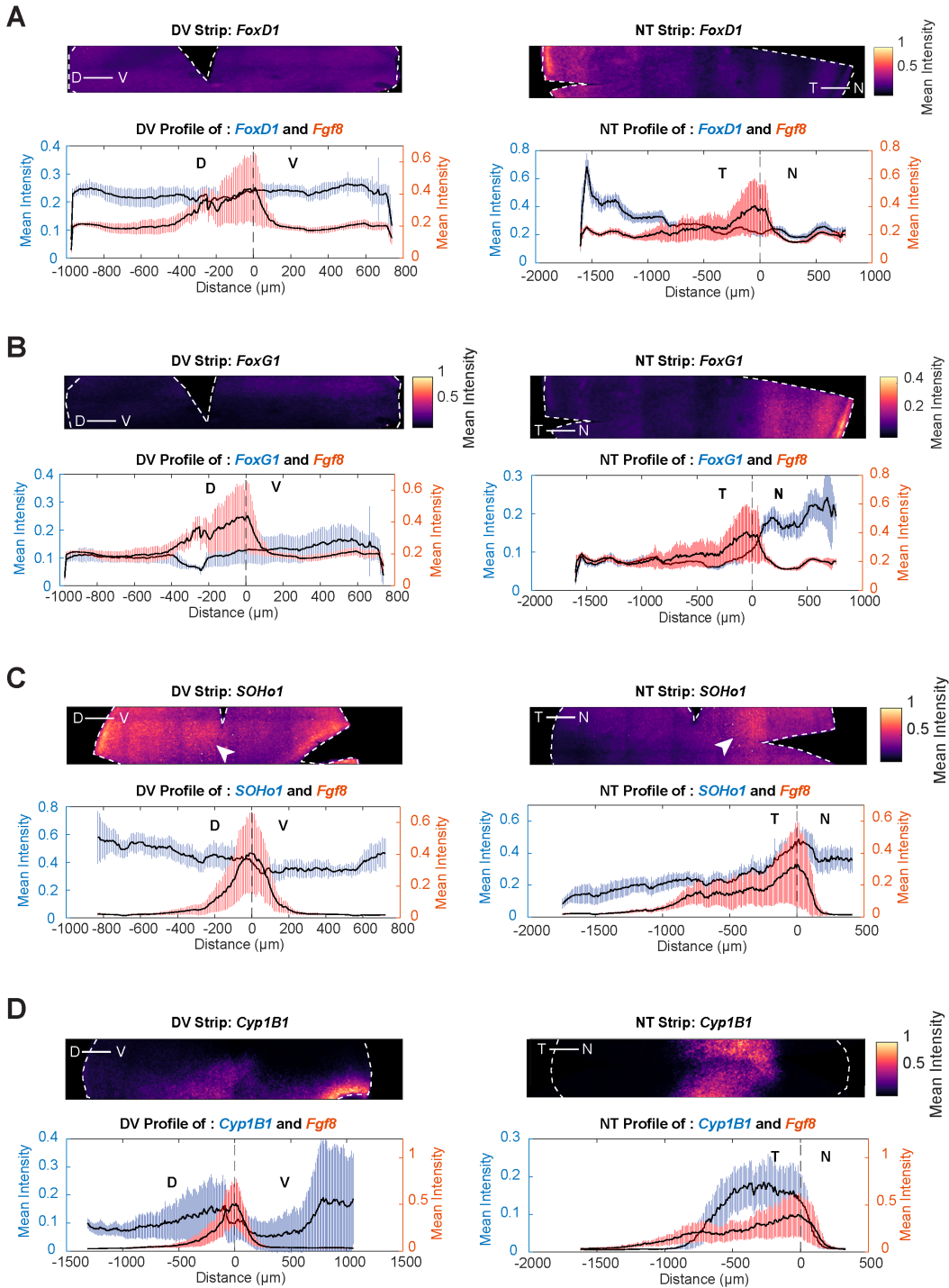

Supplement: Supplement 11 — Figure S8. Quantification of RNA-FISH signal for nasal-temporal genes relative to Fgf8 Normalized mean intensity profiles of early NT patterned genes along DV and NT axes with the origin set at the center of the HAA (Fgf8 spot). From each origin, 500 μm wide strips were extracted along the x- and y-axes, and mean intensity values were computed across 1D cross-sections to create graphs of relative gene expression. Black lines denote the mean normalized fluorescence intensity; red/blue lines represent the standard deviation (SD). DV and NT mean intensity profiles for (A) FoxD1 and Fgf8 (B) FoxG1 and Fgf8 (C) SOHo1 and Fgf8 (White arrow highlights region of peak SOHo1 expression, which aligns with the Fgf8 peak along the NT axis) (D) Cyp1B1 and Fgf8. HH, Hamburger and Hamilton; D, Dorsal; V, Ventral; N, Nasal; T, Temporal. [file media-11.pdf]

Supplementary Figure 9. Expression domains of early NT patterning genes relative to *Fgf8* at HH25/26

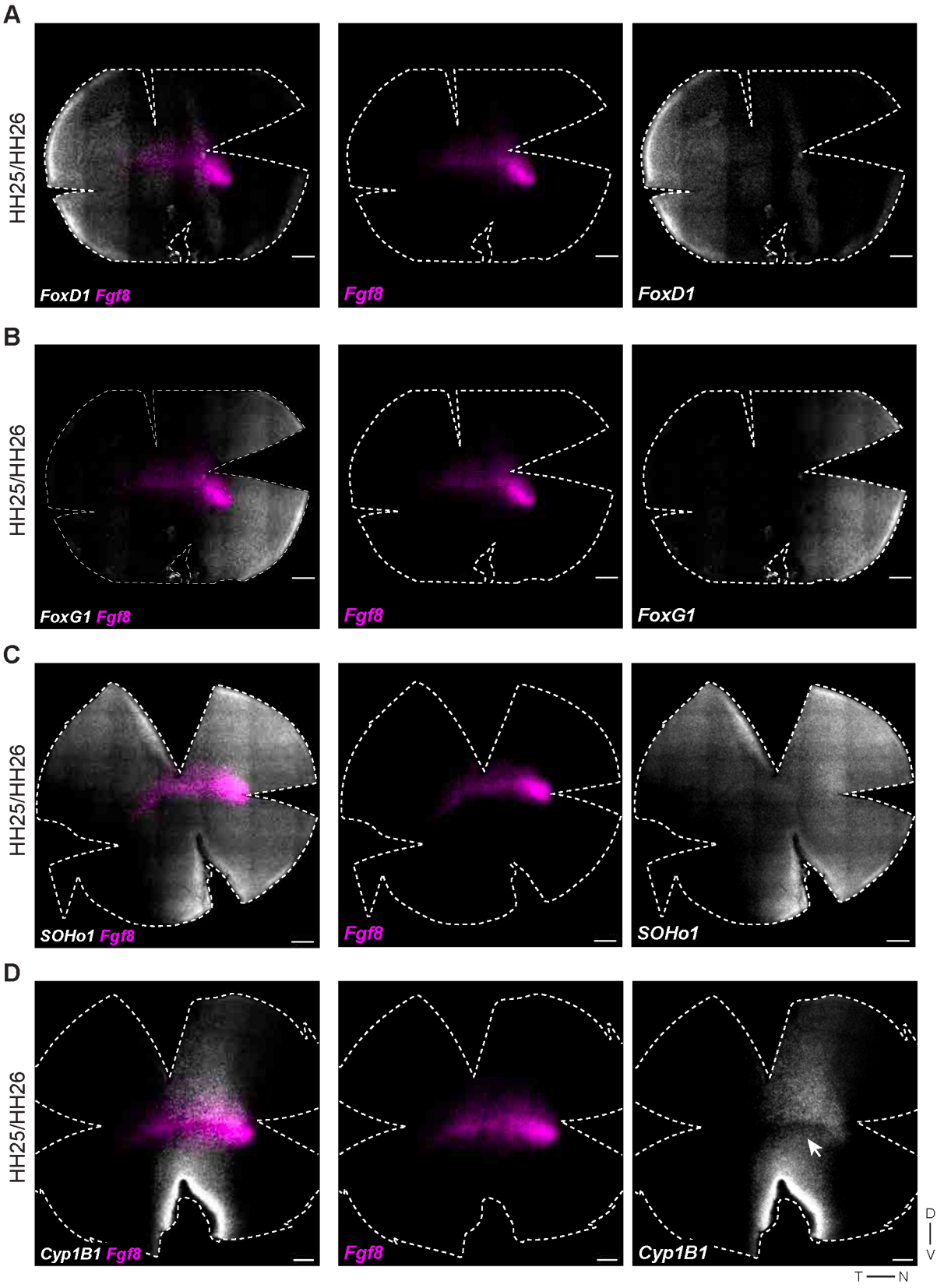

Supplement: Supplement 12 — Figure S9. Expression domains of nasal-temporal genes relative to Fgf8 at HH25/26 Multiplexed RNA-FISH was performed on HH25/HH26 retinal whole mounts for (A) Fgf8 and FoxD1; (B) Fgf8 and FoxG1; (C) Fgf8 and SOHo1; (D) Fgf8 and Cyp1B1 (White arrow highlights region of absence of Cyp1B1 expression along the equator). Each panel shows merged images, and corresponding single-channel expression images. Scale bars, 200 μm. HH, Hamburger and Hamilton; D, Dorsal; V, Ventral; N, Nasal; T, Temporal. All patterns were observed in N ≥ 3 retinas. All the images are maximum projections of confocal z-stacks. [file media-12.pdf]

Supplementary Figure 10. Expression domains of early NT patterning genes relative to *Fgf8* at HH28/29

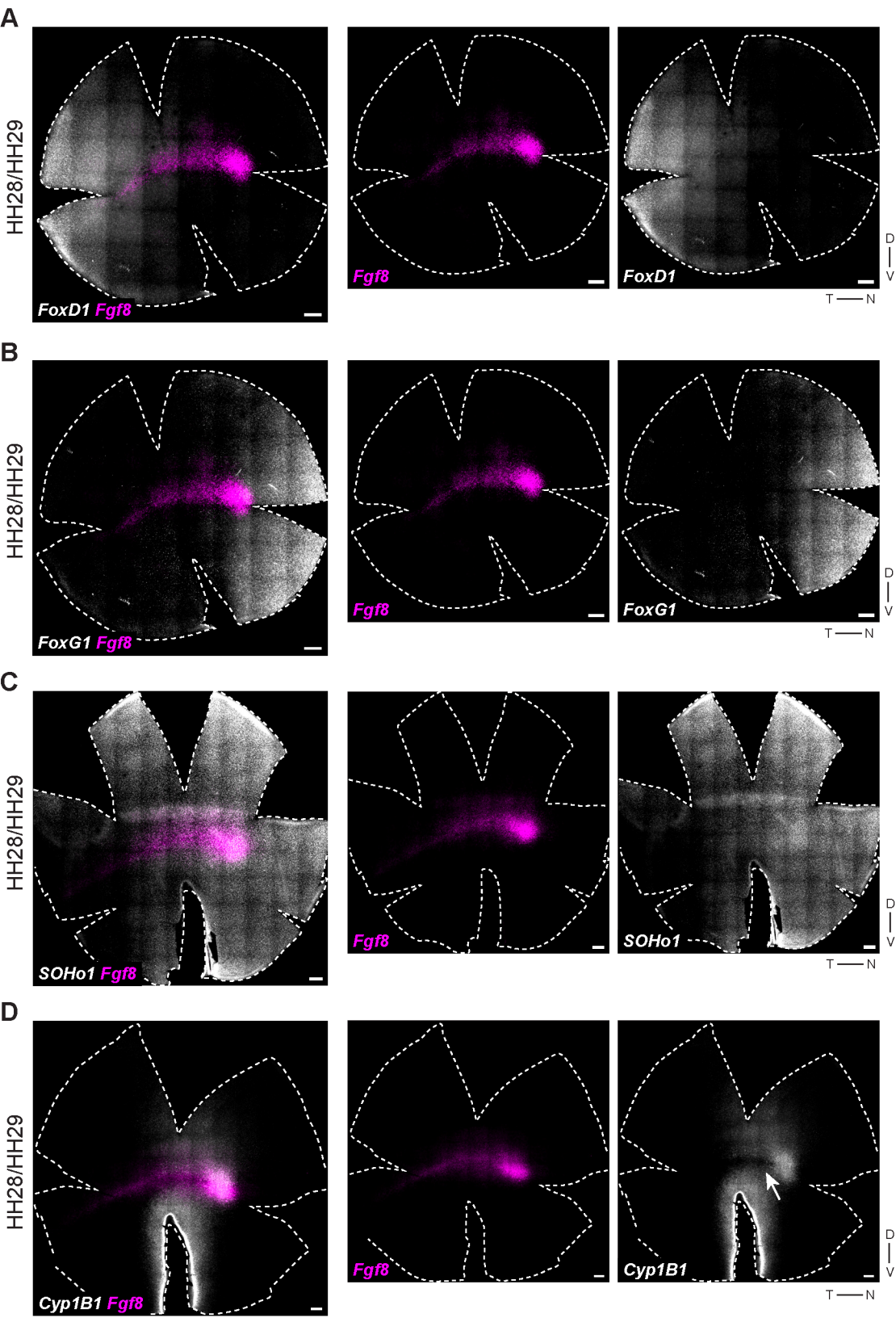

Supplement: Supplement 13 — Figure S10. Expression domains of nasal-temporal genes relative to Fgf8 at HH28/29 Multiplexed RNA-FISH was performed on HH28/HH29 retinal whole mounts for (A) Fgf8 and FoxD1; (B) Fgf8 and FoxG1; (C) Fgf8 and SOHo1; (D) Fgf8 and Cyp1B1 (White arrow highlights region of absence of Cyp1B1 expression along the equator). Each panel shows merged images, and corresponding single-channel expression images. Scale bars, 200 μm. HH, Hamburger and Hamilton; D, Dorsal; V, Ventral; N, Nasal; T, Temporal. All patterns were observed in N ≥ 3 retinas. All the images are maximum projections of confocal z-stacks. [file media-13.pdf]

Supplementary Figure 11. Quantification of RNA-FISH signal for *Bmp2* and *Visinin*

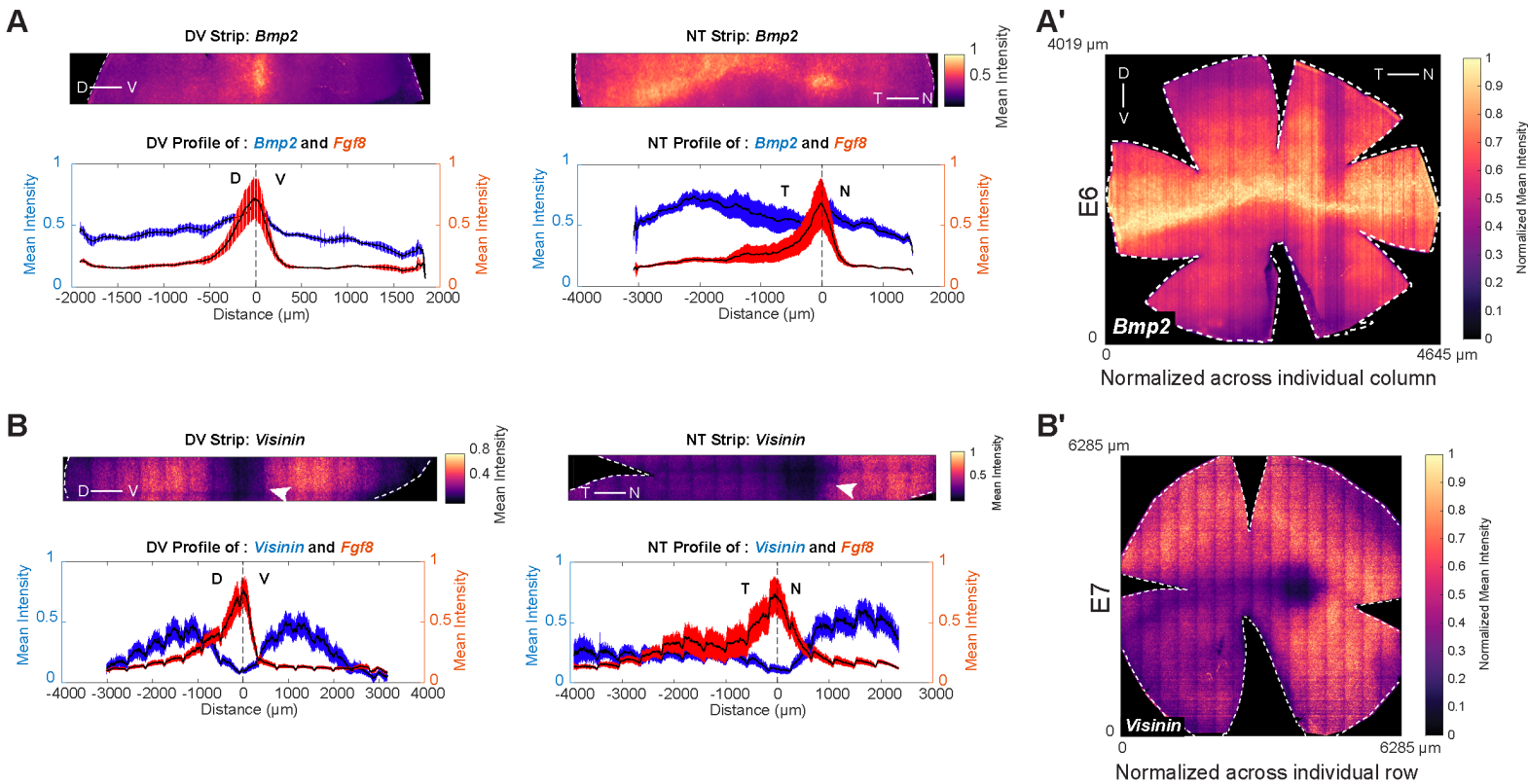

Supplement: Supplement 14 — Figure S11. Quantification of RNA-FISH signal for Bmp2 and Visinin relative to Fgf8 Multiplexed RNA-FISH was performed on (A) E6 retinal whole mounts for Fgf8 and Bmp2; and (B) E7 retinal whole mounts for Fgf8 and Visinin. Each panel shows mean intensity profiles of these genes along DV and NT axes with the origin set at the center of the Fgf8 spot. From each origin, 500 μm wide strips were extracted along the x- and y-axes, and mean intensity values were computed across 1D cross-sections to create graphs of relative gene expression. Black lines denote the mean normalized fluorescence intensity; red/blue lines represent the standard deviation (SD). (A’) Column-normalized intensity maps for Bmp2. (B’) Row-normalized intensity maps for Visinin. E, Embryonic day; D, Dorsal; V, Ventral; N, Nasal; T, Temporal. Bmp2 pattern was observed in N ≥ 3 retinas. Visinin-free spot was observed transiently and has been previously described (55). [file media-14.pdf]

Supplementary Figure 15. Grid size sensitivity analysis for 2D topographic map reconstruction

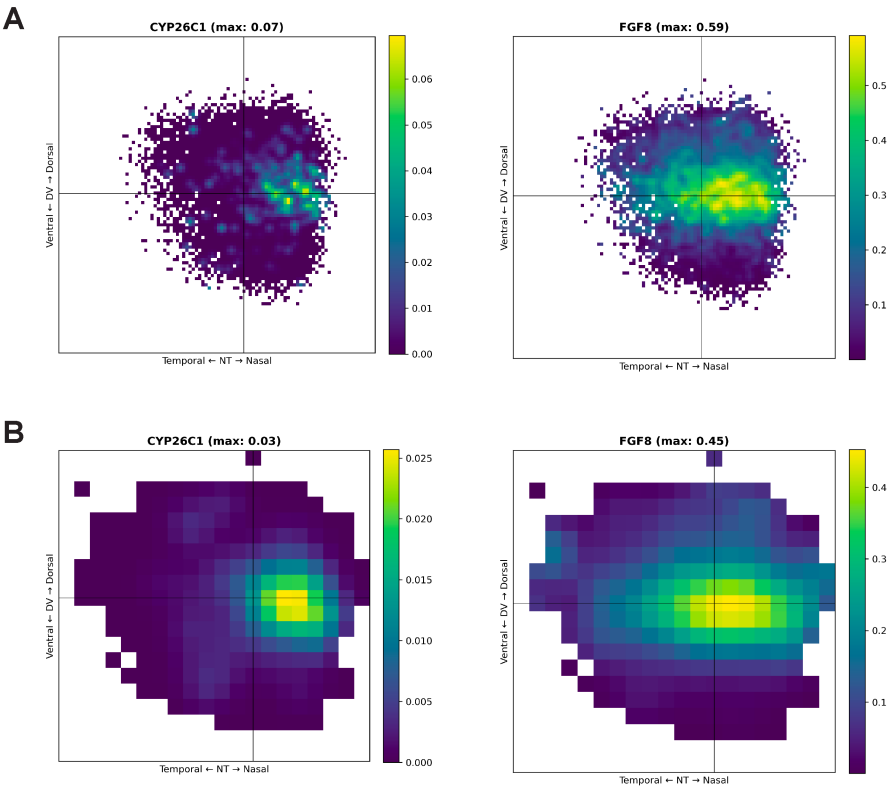

Supplement: Supplement 18 — Figure S15. Grid size sensitivity analysis for 2D topographic map reconstruction Panels show reconstructed expression maps computed using two grid resolutions: a fine grid (100 × 100 subdivisions) and a coarse grid (20 × 20 subdivisions). Each grid cell represents the mean expression value of all points falling within that spatial region. Maps are shown for Cyp26c1, which exhibits a highly specific spatial pattern but is expressed at low levels in the dataset, and Fgf8, which is both highly specific and robustly expressed. “Max” refers to the gene expression value used to normalize the upper limit of the viridis color scale. Max denotes the percentile-based upper limit of the color scale (93rd–95th percentile, species-dependent), not the absolute maximum expression. D, Dorsal; V, Ventral; N, Nasal; T, Temporal; DV.score, Dorsal-Ventral score; NT.score, Nasal-Temporal score. [file media-18.pdf]

Supplementary Figure 20. 2D topographic maps of Fgf family members in mouse and human retinal RPCs

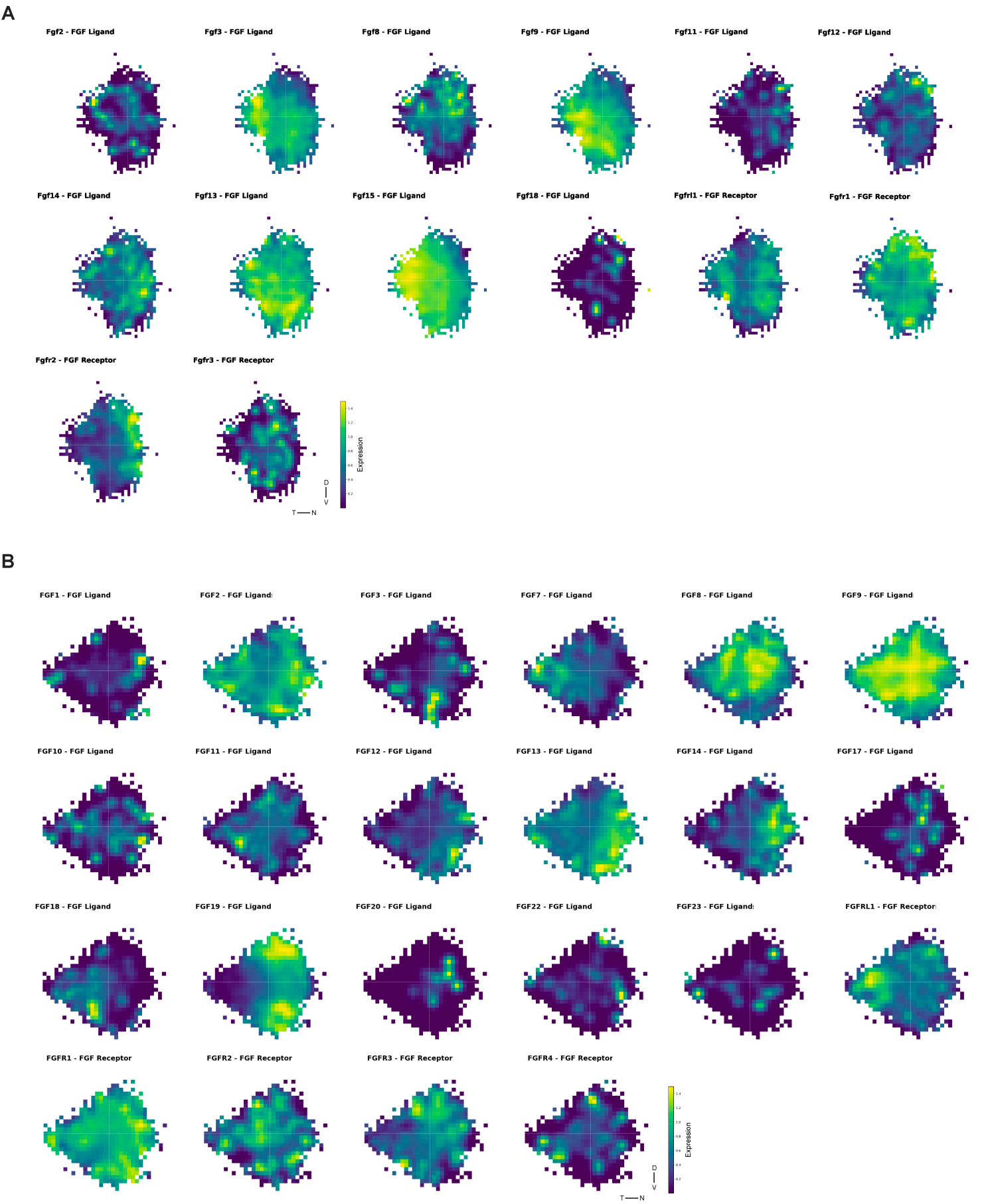

Supplement: Supplement 23 — Figure S20. 2D topographic maps of Fgf family members in mouse and human retinal RPCs 2D topographic maps of retinal gene expression of Fgf signaling ligands and receptors in retinal scRNA-seq datasets from (A) mouse and (B) human. D, Dorsal; V, Ventral; N, Nasal; T, Temporal. [file media-23.pdf]
