# Supplementary material for: Integration of in situ hybridization and scRNA-seq data provides a 2D topographical map of the developing retina across species
Source: bioRxiv. 2026 Jan 4:2026.01.04.697548. Preprint. [Version 1] doi: 10.64898/2026.01.04.697548 (PMC12776276; doi:10.64898/2026.01.04.697548)

Supplementary Figure 3. Quantification of RNA-FISH signal for *Fgf8* and RA pathway genes in retinal sections

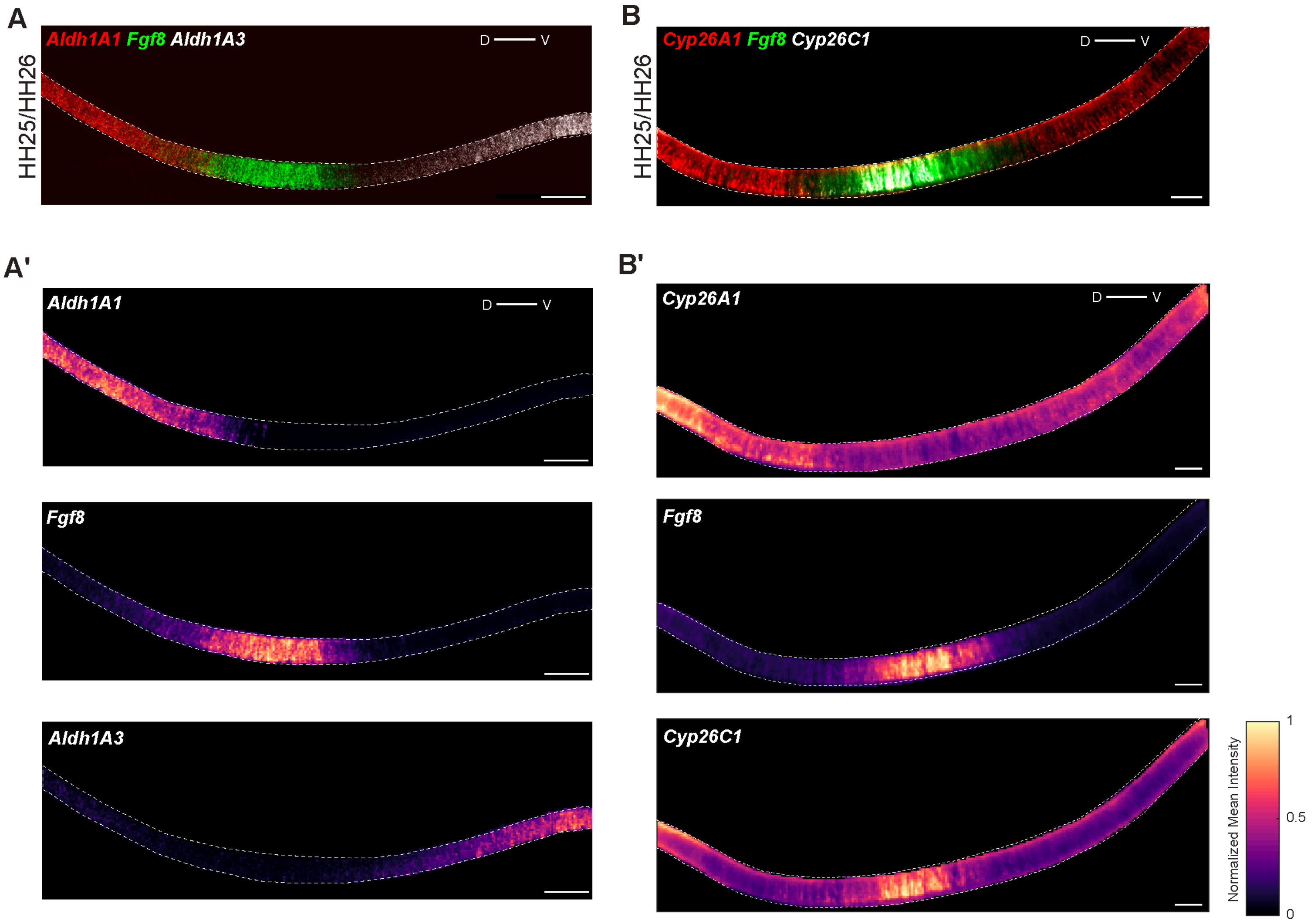

Supplement: Supplement 6 — Figure S3 Quantification of RNA-FISH signal for Fgf8 and RA pathway genes in retinal sections Multiplexed RNA-FISH hybridization on HH25/HH26 DV cross-sections through the HAA using probes for (A) Fgf8, Aldh1a1, and Aldh1a3 or (B) Fgf8, Cyp26a1, and Cyp26c1. (A’, B’) Normalized mean intensity heatmaps generated using the MATLAB pipeline. Scale bars, 100μm. HH, Hamburger and Hamilton; D, dorsal; V, ventral. [file media-6.pdf]
