# Supplementary material for: Integration of in situ hybridization and scRNA-seq data provides a 2D topographical map of the developing retina across species
Source: bioRxiv. 2026 Jan 4:2026.01.04.697548. Preprint. [Version 1] doi: 10.64898/2026.01.04.697548 (PMC12776276; doi:10.64898/2026.01.04.697548)

Supplementary Figure 13. Generation of DV score using single-cell transcriptomes from the developing chick retina

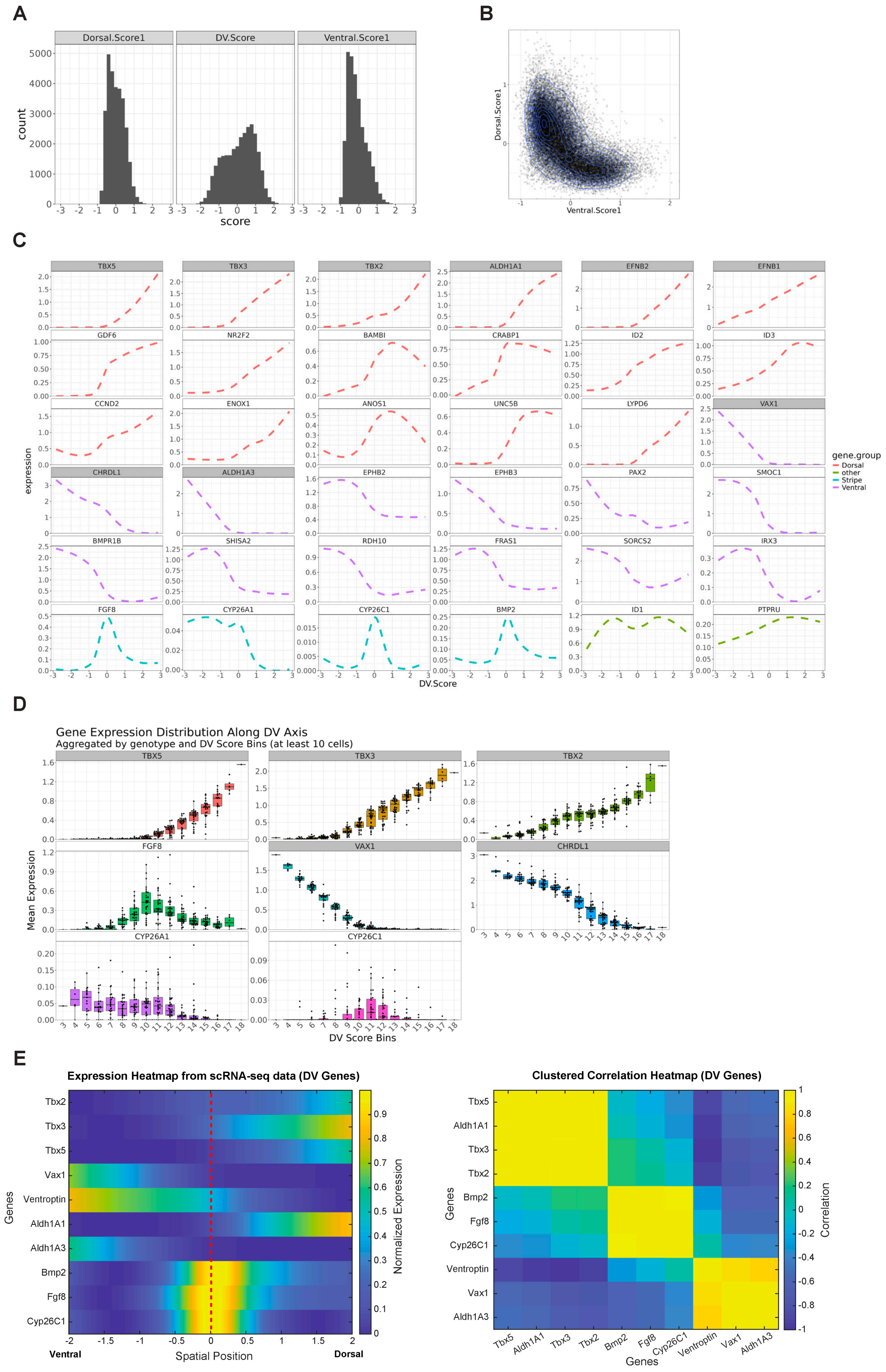

Supplement: Supplement 16 — Figure S13. Generation of DV score using single-cell transcriptomes from the developing chicken retina (A) Distribution of Dorsal, Ventral score and the subtracted composite score DV.Score. (B) Distribution of Dorsal and Ventral scores. Each point represents an individual single-cell transcriptome. (C) Distribution of gene expression along the DV.Score axis. The grayed-out genes represent the genes used for the DV score construction. (D) Binned expression pattern along the DV.Score axis, with individual points representing pseudobulked expression from individual embryos. (E) Spatial correlation analysis of selected genes from scRNA-seq data along the DV axis. Left, heatmap showing normalized expression of selected DV genes ordered by inferred DV position. Right, clustered correlation heatmap for the same genes based on pairwise Pearson correlation of their spatial expression profiles, revealing dorsal, central, and ventral gene modules. Genes are ordered according to correlation-based hierarchical clustering. The red dashed line indicates the approximate position of the developing HAA, marked at the center of the Fgf8 and Cyp26C1 expression domains. D, Dorsal; V, Ventral; DV.score, Dorsal-Ventral score. [file media-16.pdf]
