# Supplementary material for: Integration of in situ hybridization and scRNA-seq data provides a 2D topographical map of the developing retina across species
Source: bioRxiv. 2026 Jan 4:2026.01.04.697548. Preprint. [Version 1] doi: 10.64898/2026.01.04.697548 (PMC12776276; doi:10.64898/2026.01.04.697548)

Supplementary Figure 14. Generation of NT score using single-cell transcriptomes from the developing chick retina

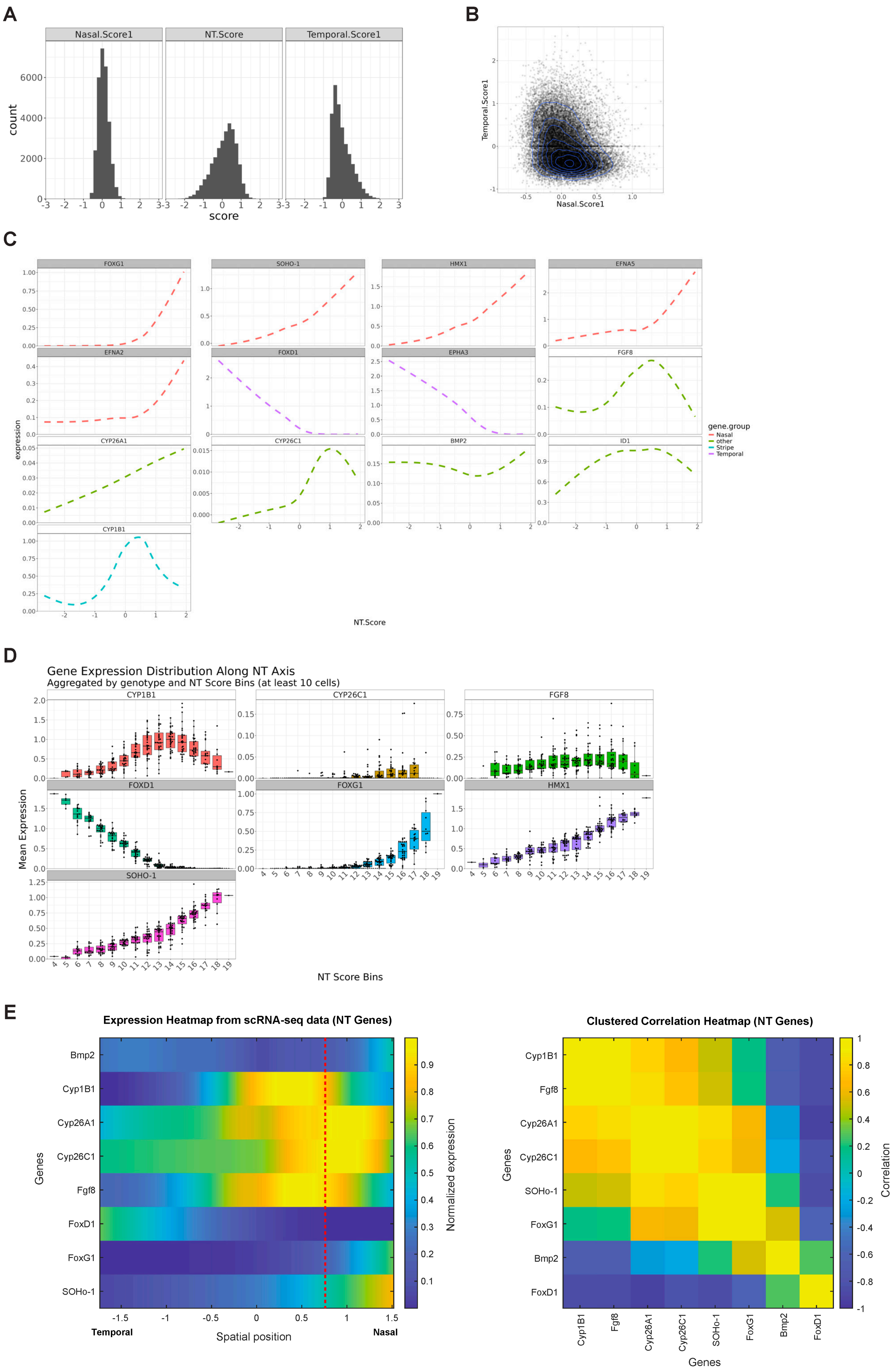

Supplement: Supplement 17 — Figure S14. Generation of NT score using single-cell transcriptomes from the developing chicken retina (A) Distribution of Nasal, Temporal score and the subtracted composite score NT.Score. (B) Distribution of Nasal, Temporal scores. Each point represents an individual single-cell transcriptome. (C) Distribution of gene expression along the NT.Score axis. The grayed-out genes represent the genes used for the NT score construction. (D) Binned expression pattern along the NT.Score axis, with individual points representing pseudobulked expression from individual embryos. (E) Spatial correlation analysis of selected genes from scRNA-seq data along the NT axis. Left, heatmap showing normalized expression of selected DV genes ordered by inferred NT position. Right, clustered correlation heatmap for the same genes based on pairwise Pearson correlation of their spatial expression profiles, revealing nasal, central, and temporal gene modules. Genes are ordered according to correlation-based hierarchical clustering. The red dashed line indicates the approximate position of the developing HAA, marked at the center of the Fgf8 and Cyp26C1 expression domains. N, Nasal; T, Temporal; NT.score, Nasal-Temporal score. [file media-17.pdf]
