# Supplementary material for: Integration of in situ hybridization and scRNA-seq data provides a 2D topographical map of the developing retina across species
Source: bioRxiv. 2026 Jan 4:2026.01.04.697548. Preprint. [Version 1] doi: 10.64898/2026.01.04.697548 (PMC12776276; doi:10.64898/2026.01.04.697548)

Supplementary Figure 16. Identification of genome-wide spatial expression patterns in the chick retina

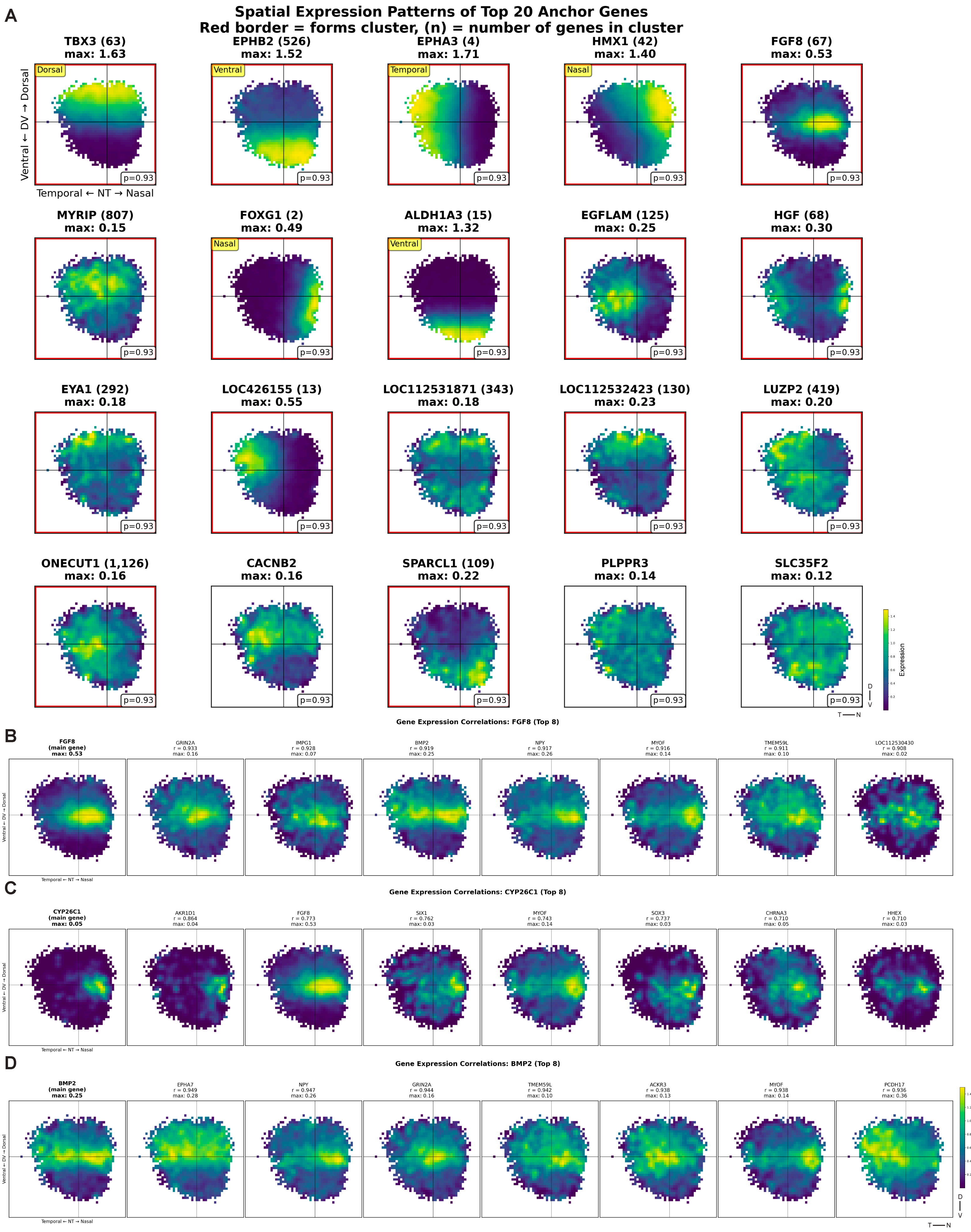

Supplement: Supplement 19 — Figure S16. Identification of spatial expression clusters in the chicken retina (A) 20 distinct spatial expression patterns (anchors) that maximize the divergence of spatial expression pattern in chicken scRNA-seq datasets. Each anchor represents a characteristic spatial profile, and genes were assigned to anchors based on similarity of their reconstructed expression patterns. Anchors that shared mutual nearest neighbors were grouped, with the associated number of genes indicated. Clusters manually annotated based on known marker gene expression are labeled and highlighted in yellow. The bottom-right square within each panel (p) shows the percentile cutoff used for visualization, where bins at or above the selected percentile are assigned the maximum color value to minimize distortion from outliers. Red border = forms cluster, (n) = number of genes in cluster. (B-D) Genes most strongly correlated with (B) Fgf8, (C) Cyp26c1, and (D) Bmp2 spatial patterns. “Max” refers to the gene expression value used to normalize the upper limit of the viridis color scale. r = Pearson correlation coefficient: perfect positive correlation (r = 1), no correlation (r = 0), perfect negative correlation (r = −1). D, Dorsal; V, Ventral; N, Nasal; T, Temporal; DV.score, Dorsal-Ventral score; NT.score, Nasal-Temporal score. [file media-19.pdf]
