# Supplementary material for: Integration of in situ hybridization and scRNA-seq data provides a 2D topographical map of the developing retina across species
Source: bioRxiv. 2026 Jan 4:2026.01.04.697548. Preprint. [Version 1] doi: 10.64898/2026.01.04.697548 (PMC12776276; doi:10.64898/2026.01.04.697548)

Supplementary Figure 17. Spatial expression patterns of genes associated with Fgf, Bmp and RA signaling pathways in the chick retina

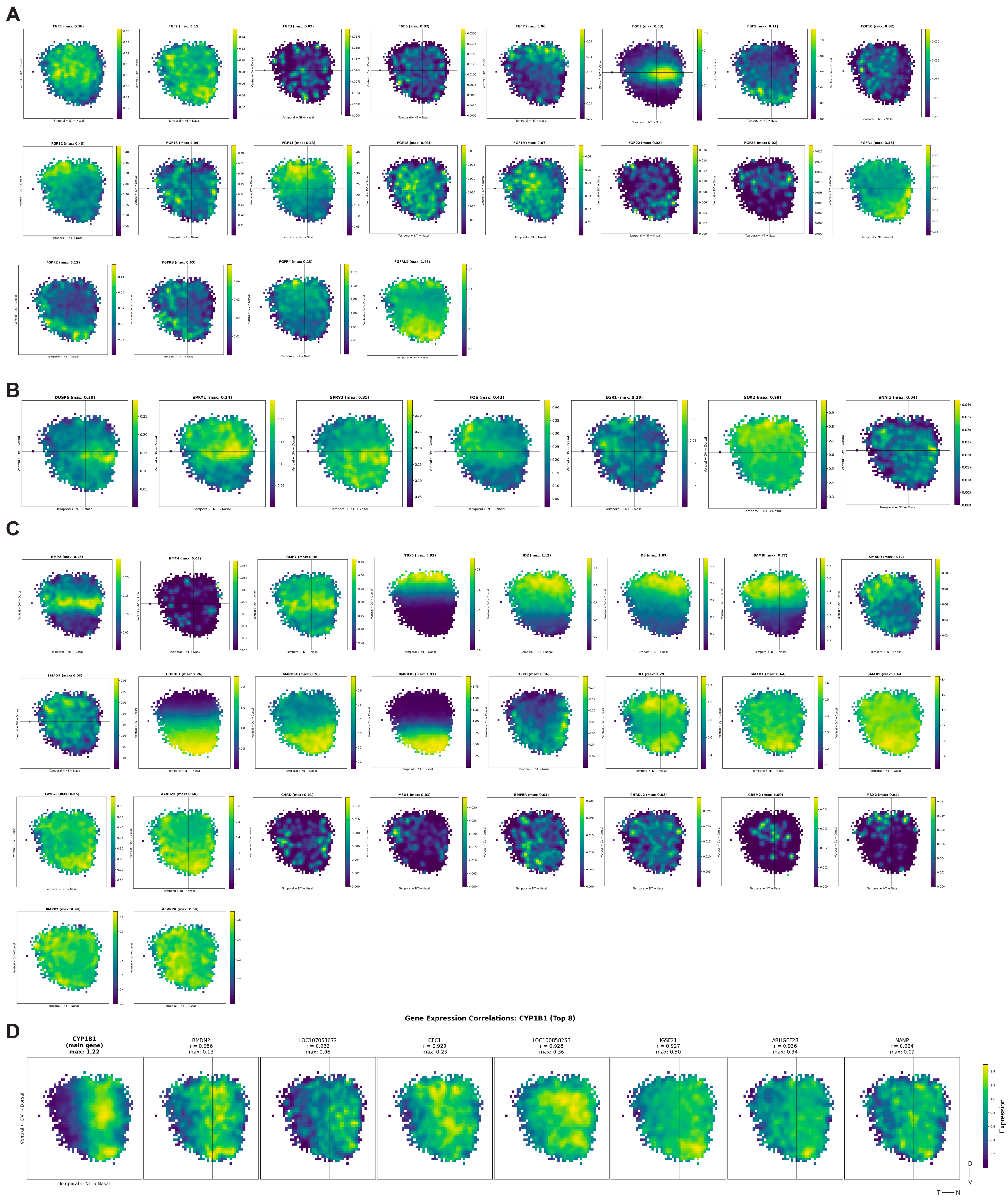

Supplement: Supplement 20 — Figure S17. Spatial expression patterns of genes associated with Fgf, Bmp and RA signaling pathways in the chicken retina 2D topographic maps of retinal gene expression of (A) Fgf signaling ligands and receptors, (B) downstream targets of Fgf8, (C) Bmp signaling pathway genes, (D) Genes most strongly correlated with the Cyp1B1 spatial pattern. “Max” refers to the gene expression value used to normalize the upper limit of the viridis color scale. r = Pearson correlation coefficient: perfect positive correlation (r = 1), no correlation (r = 0), perfect negative correlation (r = −1). D, Dorsal; V, Ventral; N, Nasal; T, Temporal; DV.score, Dorsal-Ventral score; NT.score, Nasal-Temporal score. [file media-20.pdf]
