# Supplementary material for: Integration of in situ hybridization and scRNA-seq data provides a 2D topographical map of the developing retina across species
Source: bioRxiv. 2026 Jan 4:2026.01.04.697548. Preprint. [Version 1] doi: 10.64898/2026.01.04.697548 (PMC12776276; doi:10.64898/2026.01.04.697548)

Supplementary Figure 18. Generation of DV and NT scores using single-cell transcriptomes from developing mouse and human retinas

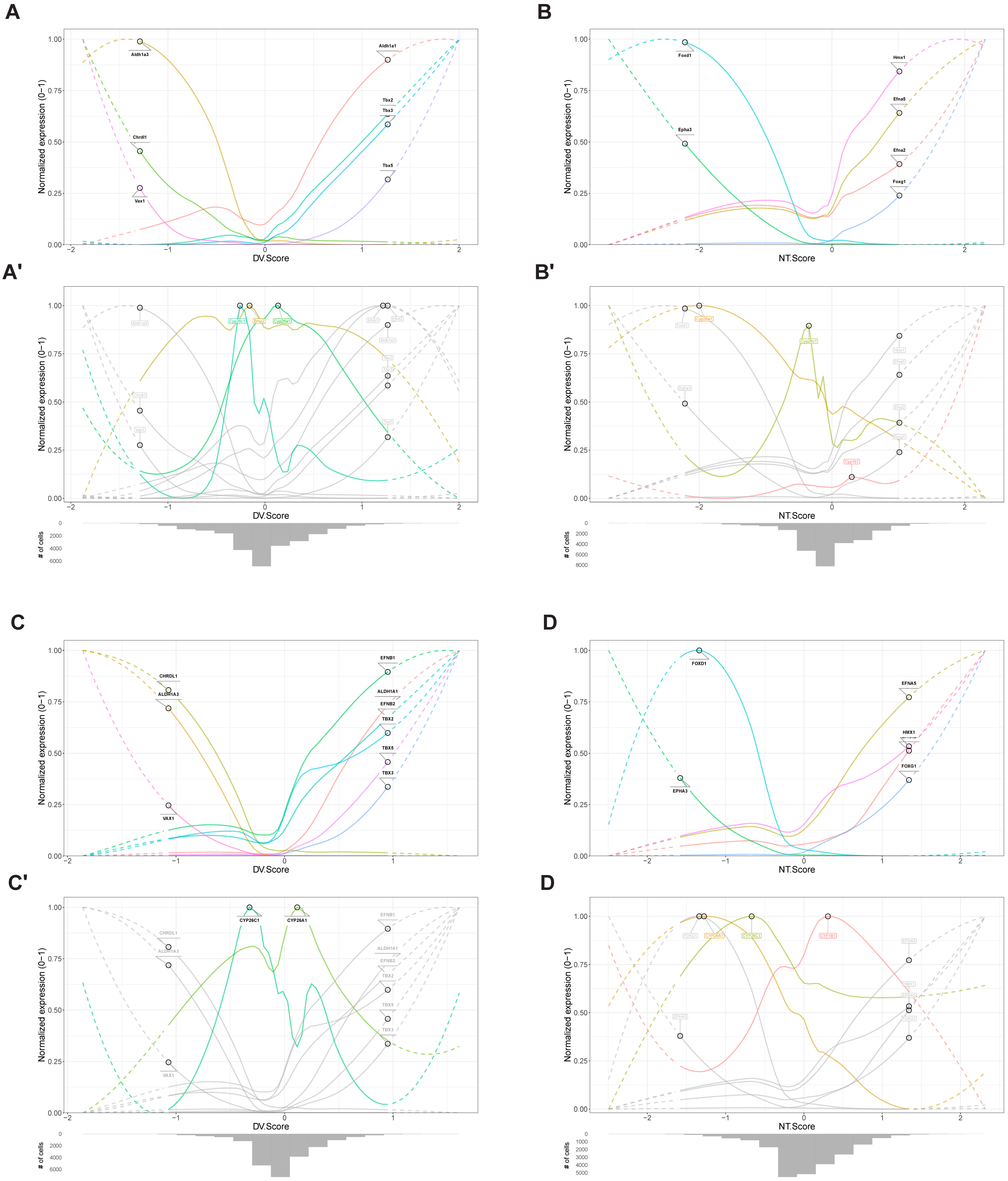

Supplement: Supplement 21 — Figure S18 Generation of DV and NT scores using single-cell transcriptomes from developing mouse and human retinas Spatial expression pattern of marker genes used to calculate the (A, C) DV.Score and (B, D) NT.score using scRNA-seq data from developing (A, B) mouse and (C, D) human retina. From the data, D, V, N and T scores were computed with the combination of {Tbx5, Tbx2, Tbx3, Aldh1a1, EfnB2, EfnB1}, {Vax1, Chrdl1, Aldh1a3}, {FoxG1, SOHo-1, Hmx1, EfnA5, EfnA2}, and {FoxD1, EphA3} respectively. Validation of the spatial reconstruction pipeline using independent genes with well-defined axial expression patterns that were not included in the scoring set; (A’, C’) Fgf8, Cyp26c1 and Bmp2 for validation along DV axis and (B’, D’) Fgf8, Cyp1B1, and Cyp26c1 for validation along NT axis. The histogram on the bottom represent coverage by aggregating all cells that contributed to the bins (total bins=50). The dashed lines represent the DV or NT axis bins that do not have more than 50 cells covered, suggesting less confidence for proper DV/NT.Score. [file media-21.pdf]
