# Supplementary material for: Integration of in situ hybridization and scRNA-seq data provides a 2D topographical map of the developing retina across species
Source: bioRxiv. 2026 Jan 4:2026.01.04.697548. Preprint. [Version 1] doi: 10.64898/2026.01.04.697548 (PMC12776276; doi:10.64898/2026.01.04.697548)

Supplementary Figure 19. Identification of clusters with spatial expression patterns in mouse and human retinas

A

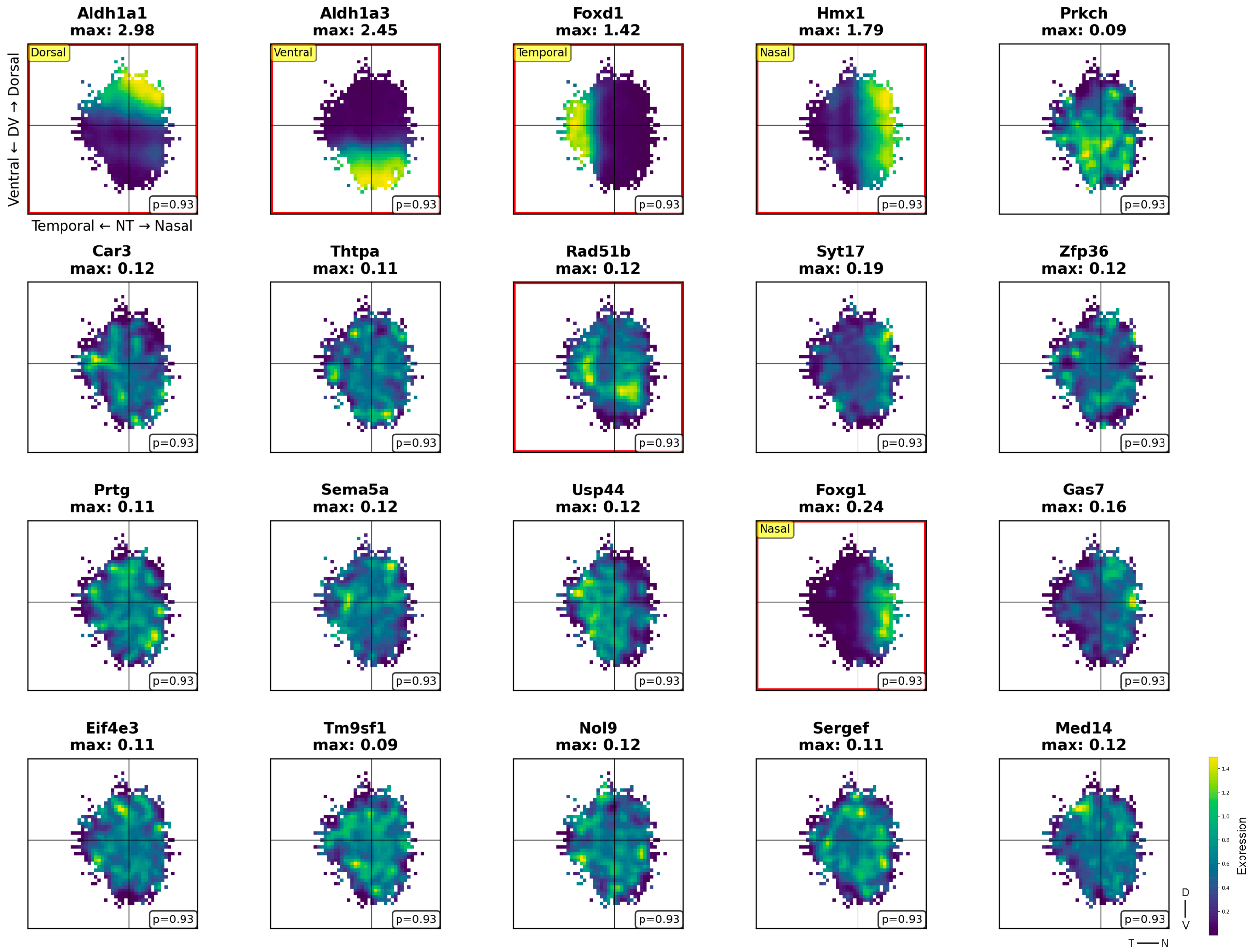

B

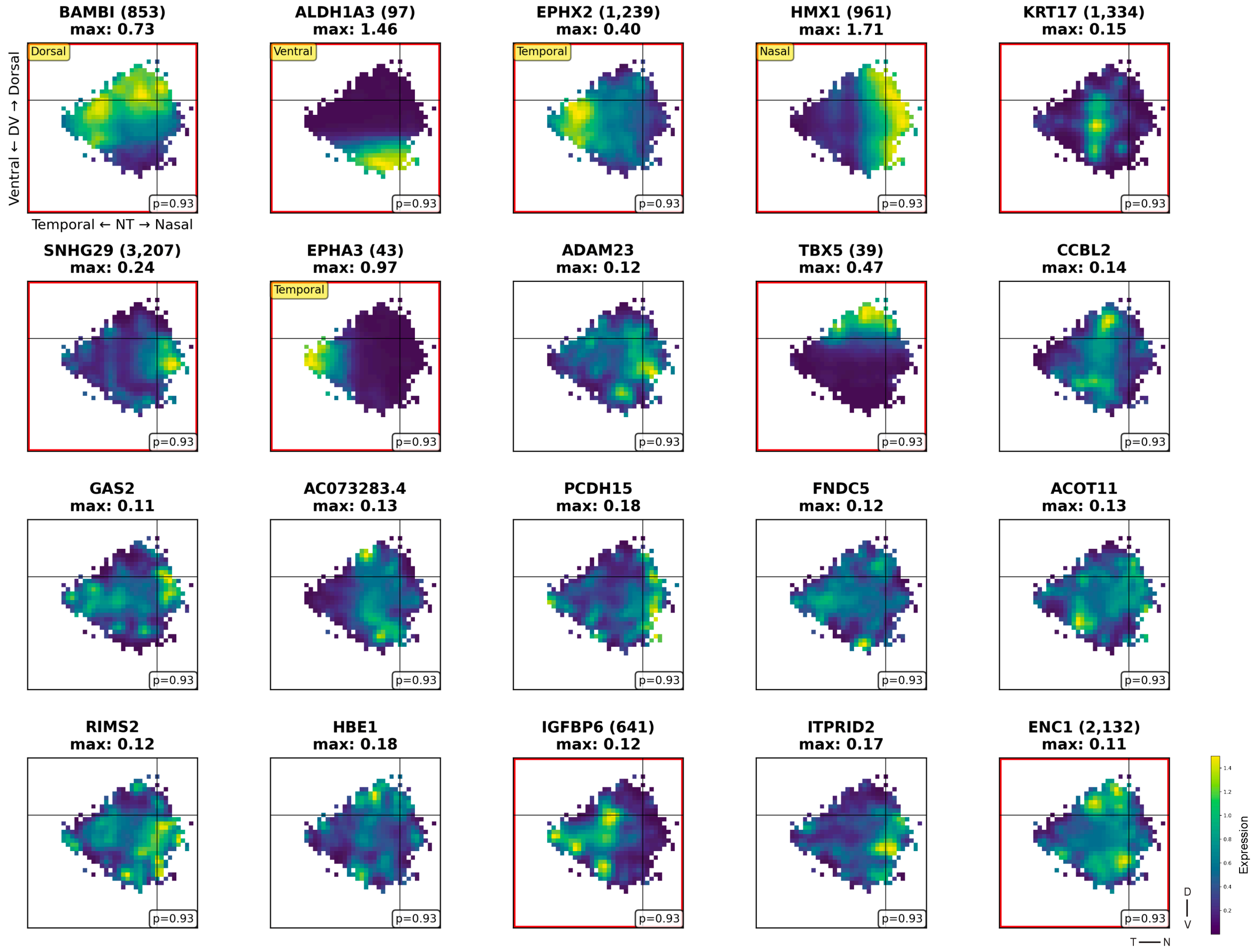

Supplement: Supplement 22 — Figure S19. Identification of clusters for spatial expression patterns in mouse and human retinas Spatial patterns (anchors) that maximize the divergence of spatial expression pattern were detected using (A) mouse and (B) human retinal scRNA-seq datasets. Anchors that have mutual neighbors - that is, genes exhibiting reciprocal similarity in their spatial expression patterns - were highlighted with the number of genes associated with the cluster (set of genes that share similar expression patterns). 6 main clusters were detected in mouse and 10 in the human dataset. A yellow label indicated manual annotation of certain clusters. The bottom right presents the percentile clip. Red border = forms cluster, (n) = number of genes in cluster. “Max” refers to the gene expression value used to normalize the upper limit of the viridis color scale. D, Dorsal; V, Ventral; N, Nasal; T, Temporal; DV.score, Dorsal-Ventral score; NT.score, Nasal-Temporal score. [file media-22.pdf]
