# Supplementary material for: Integration of in situ hybridization and scRNA-seq data provides a 2D topographical map of the developing retina across species
Source: bioRxiv. 2026 Jan 4:2026.01.04.697548. Preprint. [Version 1] doi: 10.64898/2026.01.04.697548 (PMC12776276; doi:10.64898/2026.01.04.697548)

Supplementary Figure 21. 2D topographic maps of genes related to *Fgf8* signaling pathway in mouse and human retinal RPCs

A

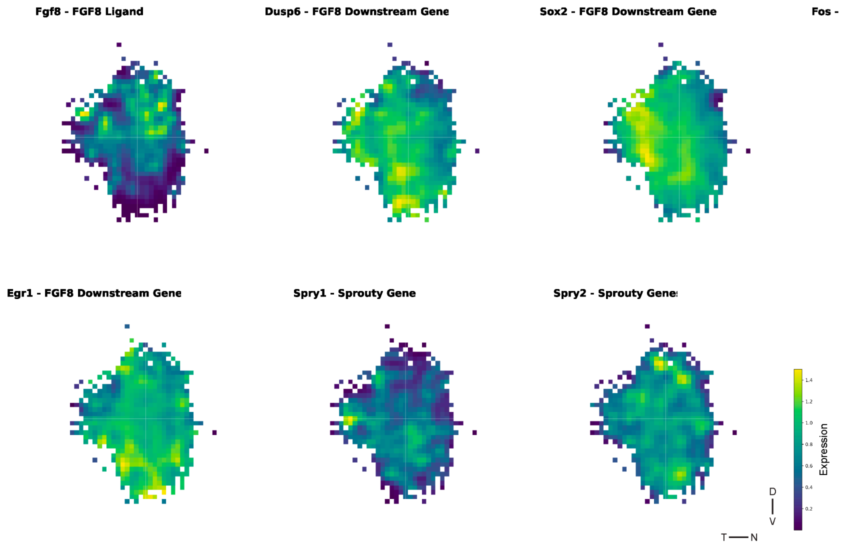

B

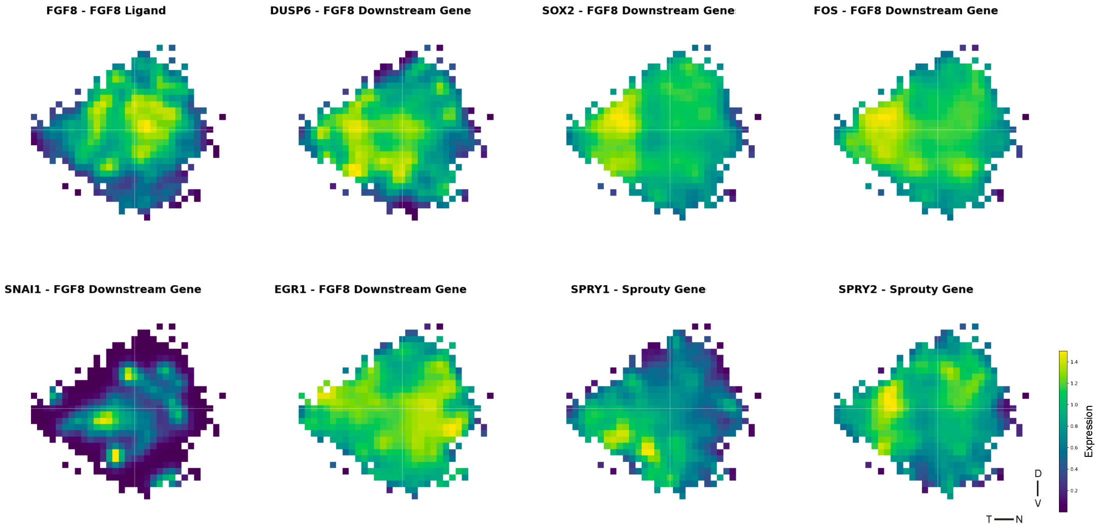

Supplement: Supplement 24 — Figure S21. 2D topographic maps of genes related to Fgf8 signaling pathway in mouse and human retinal RPCs 2D topographic maps of retinal gene expression of downstream targets of Fgf signaling in retinal scRNA-seq datasets from (A) mouse and (B) human. D, Dorsal; V, Ventral; N, Nasal; T, Temporal. [file media-24.pdf]
