# Supplementary material for: Integration of in situ hybridization and scRNA-seq data provides a 2D topographical map of the developing retina across species
Source: bioRxiv. 2026 Jan 4:2026.01.04.697548. Preprint. [Version 1] doi: 10.64898/2026.01.04.697548 (PMC12776276; doi:10.64898/2026.01.04.697548)

Supplementary Figure 22. 2D topographic maps of BMP signaling pathway components in developing mouse and human retinas

A

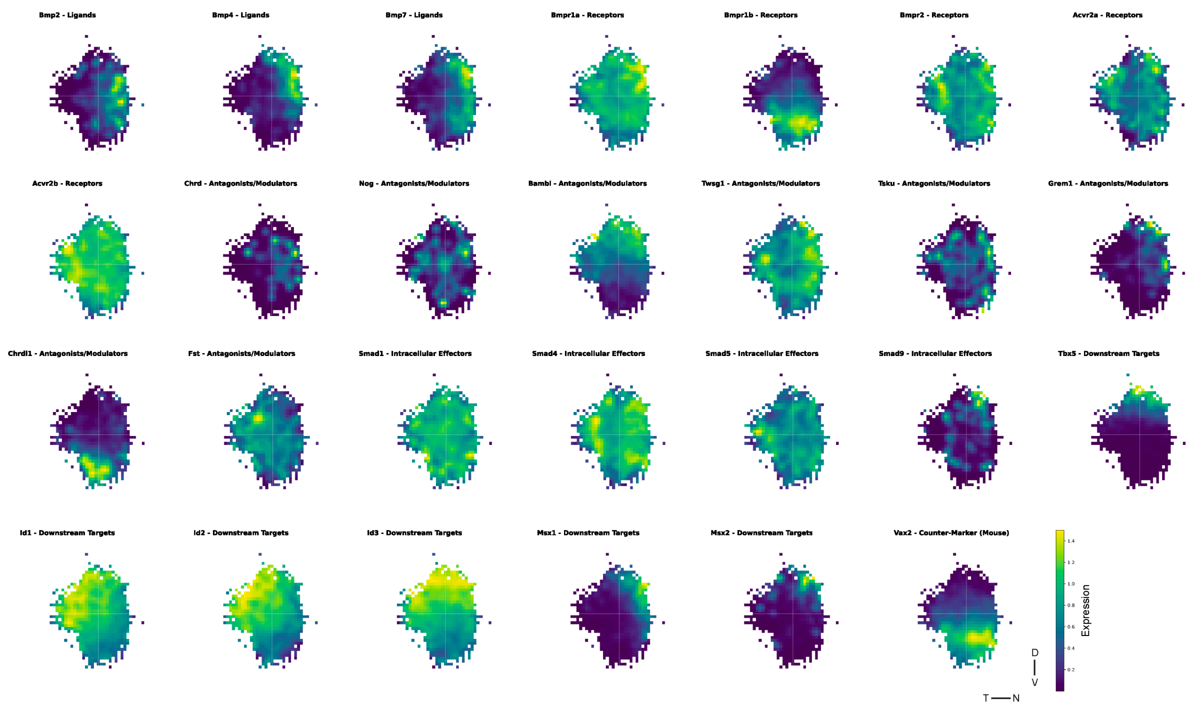

B

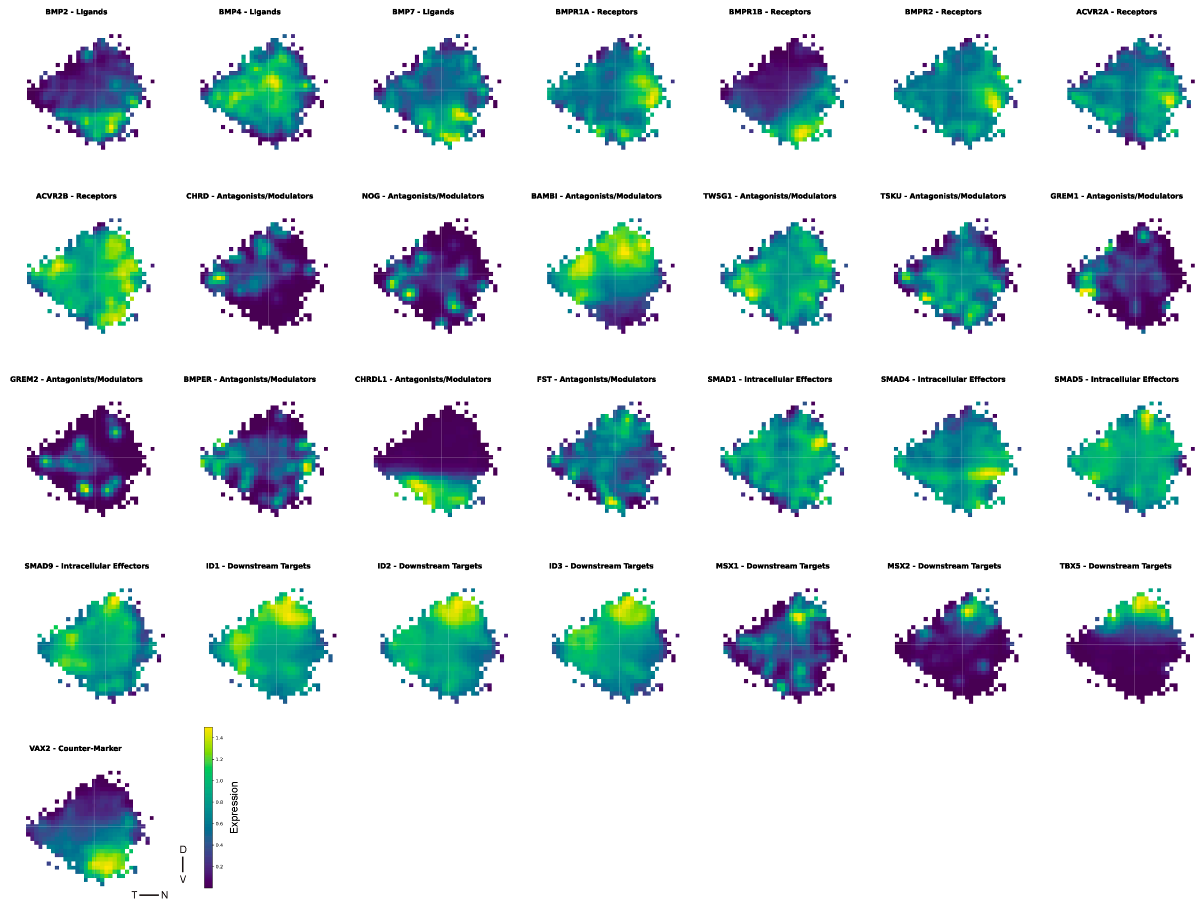

Supplement: Supplement 25 — Figure S22. 2D topographic maps of BMP signaling pathway components in developing mouse and human retinas 2D topographic maps of retinal gene expression of Bmp signaling pathway genes in retinal scRNA-seq datasets from (A) mouse and (B) human. D, Dorsal; V, Ventral; N, Nasal; T, Temporal. [file media-25.pdf]
