# Supplementary material for: Integration of in situ hybridization and scRNA-seq data provides a 2D topographical map of the developing retina across species
Source: bioRxiv. 2026 Jan 4:2026.01.04.697548. Preprint. [Version 1] doi: 10.64898/2026.01.04.697548 (PMC12776276; doi:10.64898/2026.01.04.697548)

Supplementary Figure 23. Differential gene expression analysis across different regions in the human retina

A

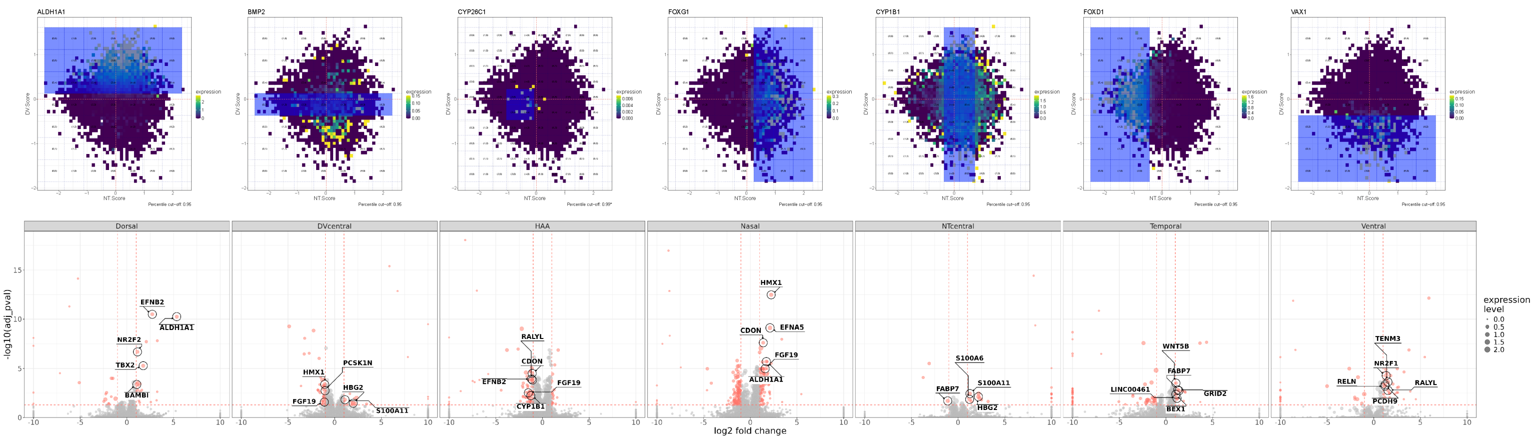

Supplement: Supplement 26 — Figure S23. Differential gene expression analysis across different regions in the human retina (A) Visual representation of virtual selection of the specific regions in the 2D topographic maps to assess differentially enriched or de-enriched genes across these regions. The 2D topographic gene expression of “region-specific marker gene” was overlayed with area bins, and the selected region was overlayed with blue. Volcano plots were generated using pseudobulked gene expression samples that are enriched or de-enriched in the selected regions. Points are scaled by average gene expression level, and genes above 2-fold expression difference between in-group and out-group and adjusted p-values less than 0.05 were colored. The top 5 enriched genes without the “LOC” prefix were highlighted. See also Supplemental Table 2. Region-specific marker genes: Aldh1a1 (Dorsal), Bmp2 (Central domain across Dorsal-Ventral axis), Cyp26c1 (HAA), FoxG1 (Nasal), Cyp1B1 (Central domain across Nasal-Temporal axis), FoxD1 (Temporal), Vax1 (Ventral). Scale bars, 200 μm. HAA, High(er) Acuity Area; N, Nasal; T, Temporal. [file media-26.pdf]
