# Supplementary material for: Integration of in situ hybridization and scRNA-seq data provides a 2D topographical map of the developing retina across species
Source: bioRxiv. 2026 Jan 4:2026.01.04.697548. Preprint. [Version 1] doi: 10.64898/2026.01.04.697548 (PMC12776276; doi:10.64898/2026.01.04.697548)

Supplementary Figure 7. Expression domains of Eph/Ephrin signaling genes in the developing chicken retina

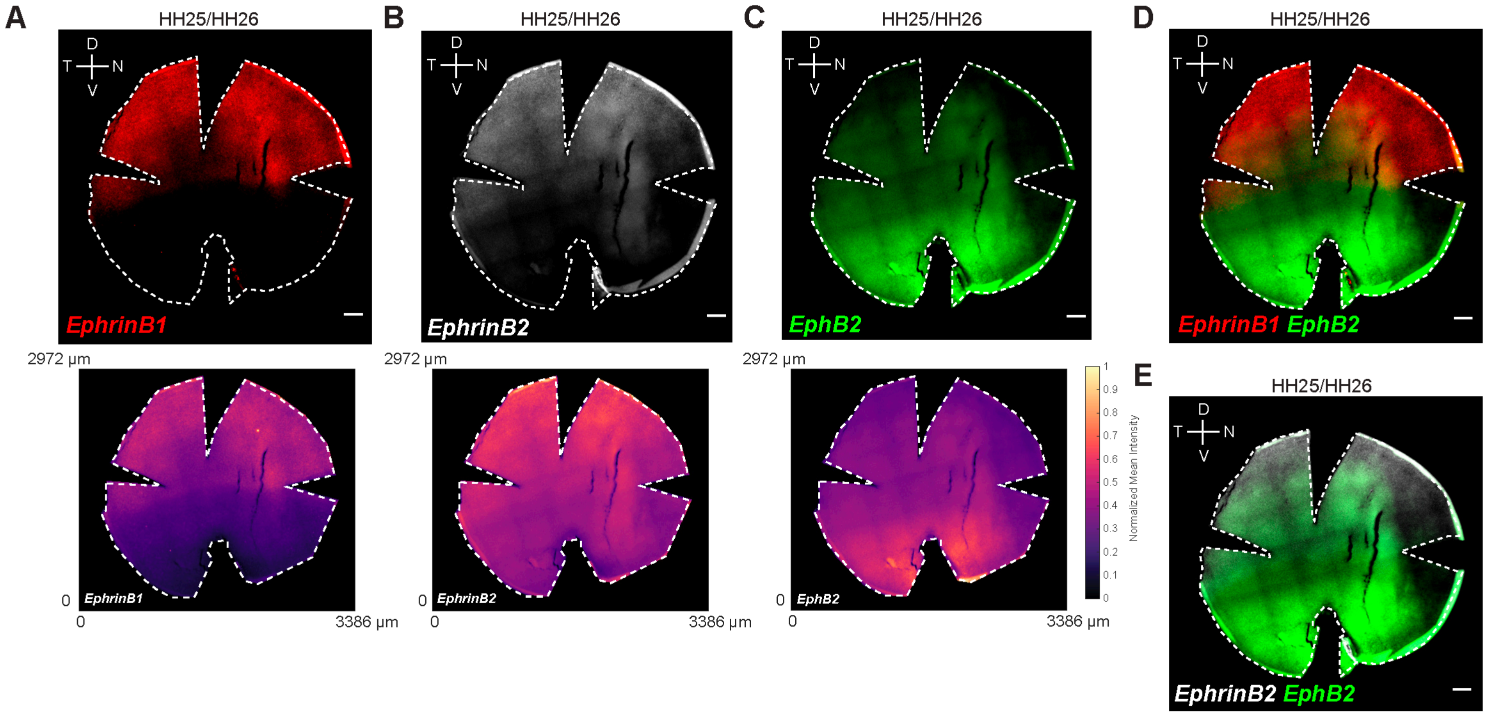

Supplement: Supplement 10 — Figure S7. Expression domains of Eph/Ephrin in the developing chicken retina Multiplexed RNA-FISH was performed on HH25/HH26 retinal whole mounts for (A) EphrinB1 (B) EphrinB2 (C) EphB2. Each panel shows single-channel expression images, and corresponding quantified intensity heatmaps. (D-E) Merged images with two channels for visualization of relative expression domains. Dorsally patterned genes (EphrinB1 and EphrinB2); Ventrally patterned genes (EphB2); Scale bars, 200 μm. HH, Hamburger and Hamilton; D, Dorsal; V, Ventral; N, Nasal; T, Temporal. All patterns were observed in N ≥ 3 retinas. All the images are widefield. [file media-10.pdf]
