# Supplementary material for: Integration of in situ hybridization and scRNA-seq data provides a 2D topographical map of the developing retina across species
Source: bioRxiv. 2026 Jan 4:2026.01.04.697548. Preprint. [Version 1] doi: 10.64898/2026.01.04.697548 (PMC12776276; doi:10.64898/2026.01.04.697548)

Supplementary Figure 12. Processing of scRNA-seq datasets from developing chick retina

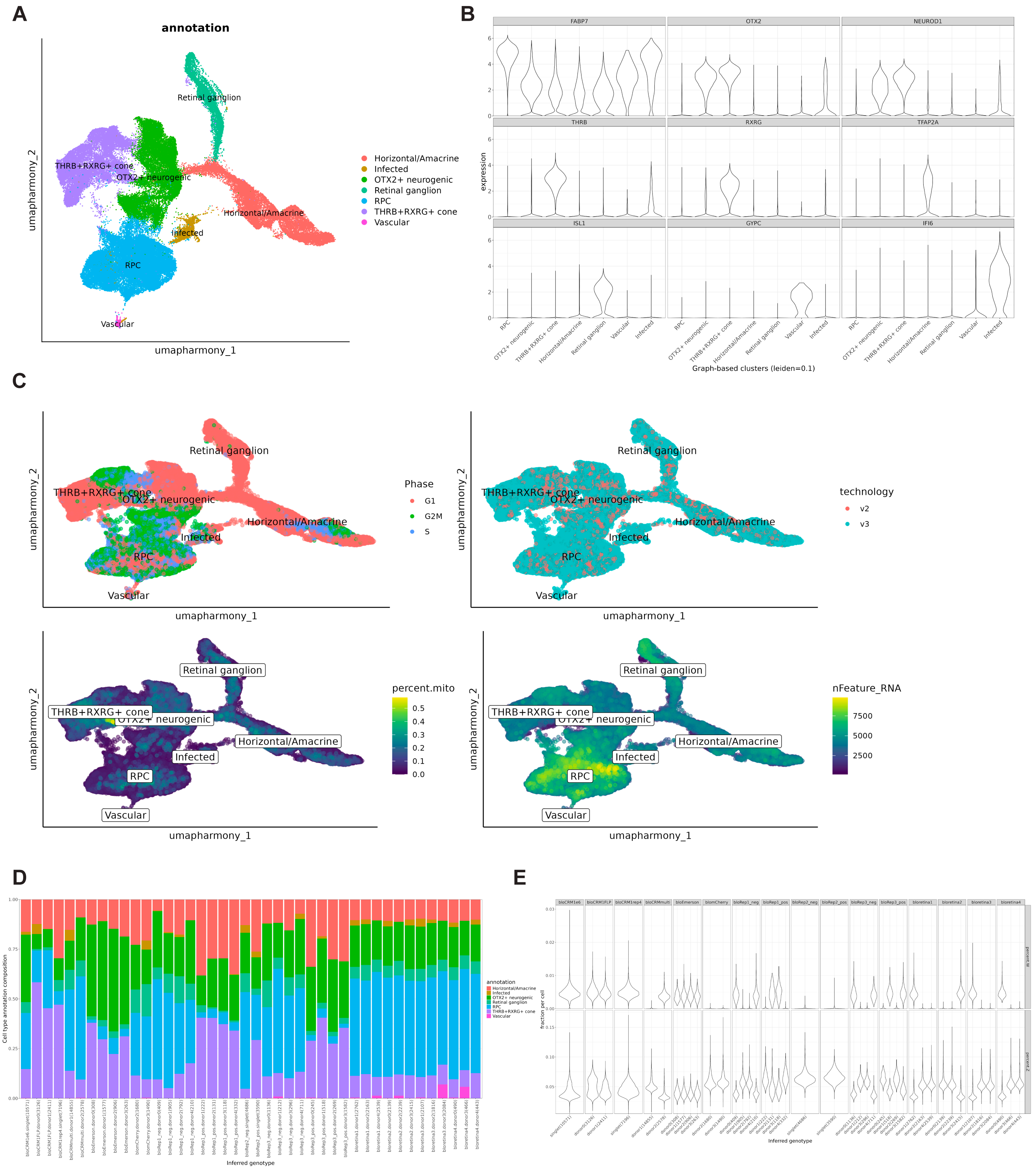

Supplement: Supplement 15 — Figure S12. Processing of scRNA-seq datasets from developing chicken retina (A) UMAP plot of major cell clusters from chicken E5–6 retinal samples. (B) Violin plot of marker gene expression across the graph-based clusters (Leiden resolution=0.1). (C) UMAP plots color coded by cell cycle phase (top left), version of 10X Chromium technology (top right), mitochondrial content (bottom left), gene count per cell (bottom right). (D) Cell type composition of samples from each embryo. (E) Violin plot of W chromosome and Z chromosome gene fraction per cell for inferred individual embryos from all libraries. Note that avian transcriptomes do not display sex chromosome dosage compensation such that females (WZ) have half the Z gene expression than males (ZZ), and males do not have W gene expression. [file media-15.pdf]
